# Engineered virus-like particle-assembled *Vegfa*-targeting Cas9 ribonucleoprotein treatment alleviates neovascularization in wet age-related macular degeneration

**DOI:** 10.1186/s13059-025-03774-5

**Published:** 2025-10-09

**Authors:** Jun Wu, Hyewon Jang, Hyunjong Kwak, Minchae Son, Weiyan Jiang, Hye-Yeon Hwang, Dong Hyun Jo, Daesik Kim, Hyongbum Henry Kim, Jeong Hun Kim

**Affiliations:** 1 Fight against Angiogenesis-Related Blindness (FARB) Laboratory, Biomedical research institute, Seoul National University Hospital, Seoul, 03082 Republic of Korea; 2 Department of Biomedical Sciences, Seoul National University College of Medicine, Seoul, 03080 Republic of Korea; 3 Department of Pharmacology, Yonsei University College of Medicine, Seoul, 03722 Republic of Korea; 4 Department of Precision Medicine, Sungkyunkwan University School of Medicine, Suwon, 16419 Republic of Korea; 5 Department of Anatomy and Cell Biology, Seoul National University College of Medicine, Seoul, 03080 Republic of Korea; 6 Brain Korea 21 Plus Project for Medical Sciences, Yonsei University College of Medicine, Seoul, 03722 Republic of Korea; 7 Center for Nanomedicine, Institute for Basic Science (IBS), Seoul, 03722 Republic of Korea; 8 Graduate Program of Nano Biomedical Engineering (NanoBME), Advanced Science Institute, Yonsei University, Seoul, 03722 Republic of Korea; 9 Woo Choo Lee Institute for Precision Drug Development, Yonsei University College of Medicine, Seoul, 03722 Republic of Korea; 10 Institute for Immunology and Immunological Diseases, Yonsei University College of Medicine, Seoul, 03722 Republic of Korea; 11 Won-Sang Lee Institute for Hearing Loss, Yonsei University College of Medicine, Seoul, 03722 Republic of Korea; 12 Department of Ophthalmology, Seoul National University College of Medicine, Seoul, 03080 Republic of Korea; 13 Global Excellence Center for Gene & Cell Therapy (GEC-GCT), Seoul National University Hospital, Seoul, 03080 Republic of Korea; 14 Institute of Reproductive Medicine and Population, Seoul National University College of Medicine, Seoul, 03080 Republic of Korea

**Keywords:** Engineered virus-like particles, Cas9 ribonucleoprotein, Age-related macular degeneration, Laser-induced choroidal neovascularization, Vascular endothelial growth factor

## Abstract

**Background:**

Age-related macular degeneration, particularly the wet form, is a leading cause of vision loss, characterized by vascular endothelial growth factor A (VEGFA) overproduction. Engineered virus-like particles (eVLPs) combine the efficiency of viral systems with the transient nature of non-viral platforms to offer a potential solution for delivering VEGFA-targeting genome editing enzymes in a safe and efficient manner. Here, we investigate the therapeutic efficacy of eVLPs for transient delivery of *Vegfa*-targeting Cas9 ribonucleoprotein in a laser-induced choroidal neovascularization mouse model of wet age-related macular degeneration.

**Results:**

We find that Cas9-eVLPs enables efficient intracellular delivery in vitro, achieving up to 99% insertion and deletion frequency at *Vegfa* target locus and significant VEGFA protein downregulation in NIH/3T3 cells. A single subretinal injection of Cas9-eVLPs into the mouse retinal pigment epithelium effectively disrupts *Vegfa* expression, achieving an average indel efficiency of 16.7%. Compared to control groups, the laser-induced choroidal neovascularization mouse model exhibits significantly reduced choroidal neovascularization formation following Cas9-eVLPs intervention, and decreased VEGFA protein levels are detected in the retinal pigment epithelium. Furthermore, the retinal anatomical and functional toxicity are not affected after treatment.

**Conclusions:**

eVLPs exhibit the potential as a safe and efficient delivery platform for Cas9 ribonucleoproteins, achieving precise *Vegfa* downregulation and significant reduction in choroidal neovascularization in a mouse model of wet age-related macular degeneration. With transient delivery of gene editing enzymes, high editing efficiency, and minimal risk of genomic integration, eVLPs present a promising alternative to conventional delivery systems for advancing genome editing therapies in retinal diseases.

**Graphical Abstract:**

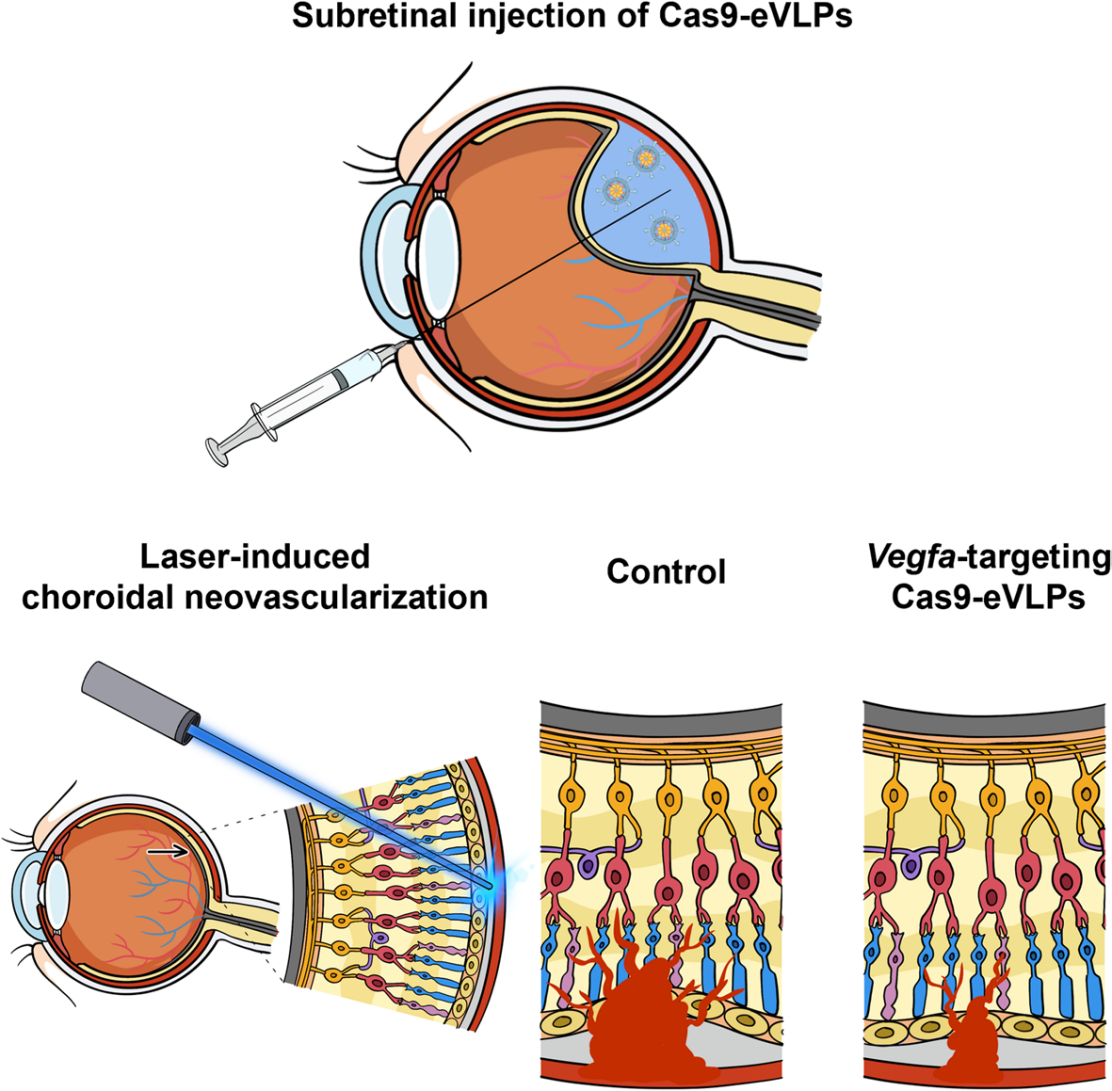

**Supplementary Information:**

The online version contains supplementary material available at 10.1186/s13059-025-03774-5.

## Background

Age-related macular degeneration (AMD) is a disease that affects the macular region of the retina, causing progressive loss of central vision among the older people [1]. Approximately 196 million people are diagnosed with AMD, a number that is expected to increase to 288 million by 2040. Thus, AMD poses considerable human and economic burdens that urgently require solutions [2]. Clinically, AMD is classified into dry and wet forms [1, 3]. Dry AMD is characterized by the gradual accumulation of drusen deposits and progressive degeneration of the retinal pigment epithelium (RPE), ultimately leading to geographic atrophy [1, 3, 4]. Wet AMD, also known as neovascular AMD, is characterized by abnormal growth of choroidal neovascularization, primarily driven by the overexpression of vascular endothelial growth factor A (VEGFA) [5]. This understanding has led to the development of anti-VEGF therapies, such as monoclonal antibodies and fusion proteins, which effectively slow disease progression and improve visual outcomes [6–8]. However, the treatment burden of frequent intravitreal injections, high costs, and the risk of treatment resistance or adverse effects highlight the need for innovative approaches that target VEGFA with greater sustainability [9–11].

The advent of third-generation genome editing systems, namely the CRISPR/Cas9, has ushered in a new era of genomic medicine, offering transformative benefits to countless patients [12]. This technology enables the precise editing of DNA sequences and can help to tackle a broad spectrum of genetic diseases and acquired conditions. For example, in individuals without pathogenic mutations, the downregulation of specific genes is crucial for therapeutic intervention, and CRISPR/Cas9 can effectively downregulate a target gene to achieve a desired effect with a one-time treatment [13, 14]. In addition, base and prime editors can directly and precisely modify mutated DNA sequences in individuals with disease-causing mutations [15–17]. To fully realize the promising potential of therapeutic genome editing tools and ensure the efficacy and safety of gene editing interventions, the precise delivery of gene editing enzymes to target sites must be ensured. At present, despite the widespread use of conventional viral and non-viral carriers for this purpose, both vector types face limitations.

Adeno-associated viruses (AAVs) are the leading viral vector to deliver gene editing cargo, owing to their widespread tissue tropism and state-of-the-art ability to overcome barriers in vivo [18]. Encouraging outcomes from preclinical and clinical investigations have underscored the efficacy of AAV-based genome editing, highlighting its potential for treating inherited as well as acquired conditions [19–24]. However, the suitability of viral vectors for therapeutic applications is limited by the risk of their neutralization and adverse immunogenicity, including in the eye, which is known as an immune-privileged tissue [25]. In addition, the possibility that viral DNA integrated into specific integration sites on chromosomes can remain latent in the host genome is of concern [25]. Lipid nanoparticles (LNPs) are a prevalent and favored platform for delivering therapeutic gene-editing agents as non-viral vectors. The development of lipo-nanomedicine for CRISPR-mediated delivery has several advantages, namely it is relatively transient, allows for repeated dosing, and mitigates safety concerns [12]. However, LNPs are considered less proficient at loading macromolecular cargo. The delivery of ribonucleoproteins (RNPs) using LNPs is less effective than that of their nucleic acid-containing counterparts [26]. Achieving therapeutic gene editing in tissues outside the liver through LNPs delivery continues to pose considerable challenges, because ApoE binding to LNPs passively directs them to accumulate in the liver [27, 28]. In addition, the ionizable lipid remained detectable in the blood stream for up to 1 week [29]. Immunogenicity is primarily driven by the ionizable lipids themselves, which trigger cytokine release and subsequent inflammatory responses [30]. Overall, a new generalizable strategy for delivering genomic cargo is needed.

Virus-like particles (VLPs) are promising vehicles for delivering genome-editing components [31, 32]. VLPs are structurally and visually similar to live viruses but lack some or all of the viral genome [33]. They retain an internal cavity that can be used to deliver biological materials. VLPs are structurally variable, contributing to their range of functions, and can be categorized as enveloped types containing host cell membranes, non-enveloped forms made of capsid proteins alone, and engineered chimeric versions carrying additional antigens [33]. Initially, VLP-based approaches for delivering gene editing agents have achieved only moderate editing efficiencies, with limited in vivo validation of their therapeutic effectiveness [34–36]. However, Banskota et al. discovered that engineered VLPs (eVLPs), a retroviral scaffold envelope supporting VLPs, effectively overcame specific molecular bottlenecks related to cargo packaging and release, showing promising results in delivering RNPs to the brain, liver, and retina in mice [37]. Thus, eVLPs have rapidly emerged as an attractive delivery platform.

The laser-induced choroidal neovascularization (LI-CNV) mouse model is widely used to study the pathogenesis of wet AMD, as it reflects key pathological features, including choroidal neovascularization and VEGFA overproduction [38, 39]. In the present study, we aimed to assess the efficacy of the eVLP system for delivering Cas9 RNPs in mouse RPE. After a single subretinal injection of *Vegfa*-targeting Cas9-eVLPs, we observed significant disrupted *Vegfa* expression and a reduction in the choroidal neovascularization. No anatomical or functional safety concerns were identified. These results highlight the potential of eVLPs as a robust and versatile RNP delivery system for therapeutic genome editing in retinal diseases.

## Results

### eVLP system demonstrates exceptional delivery performance in vitro

**Fig. 1:**
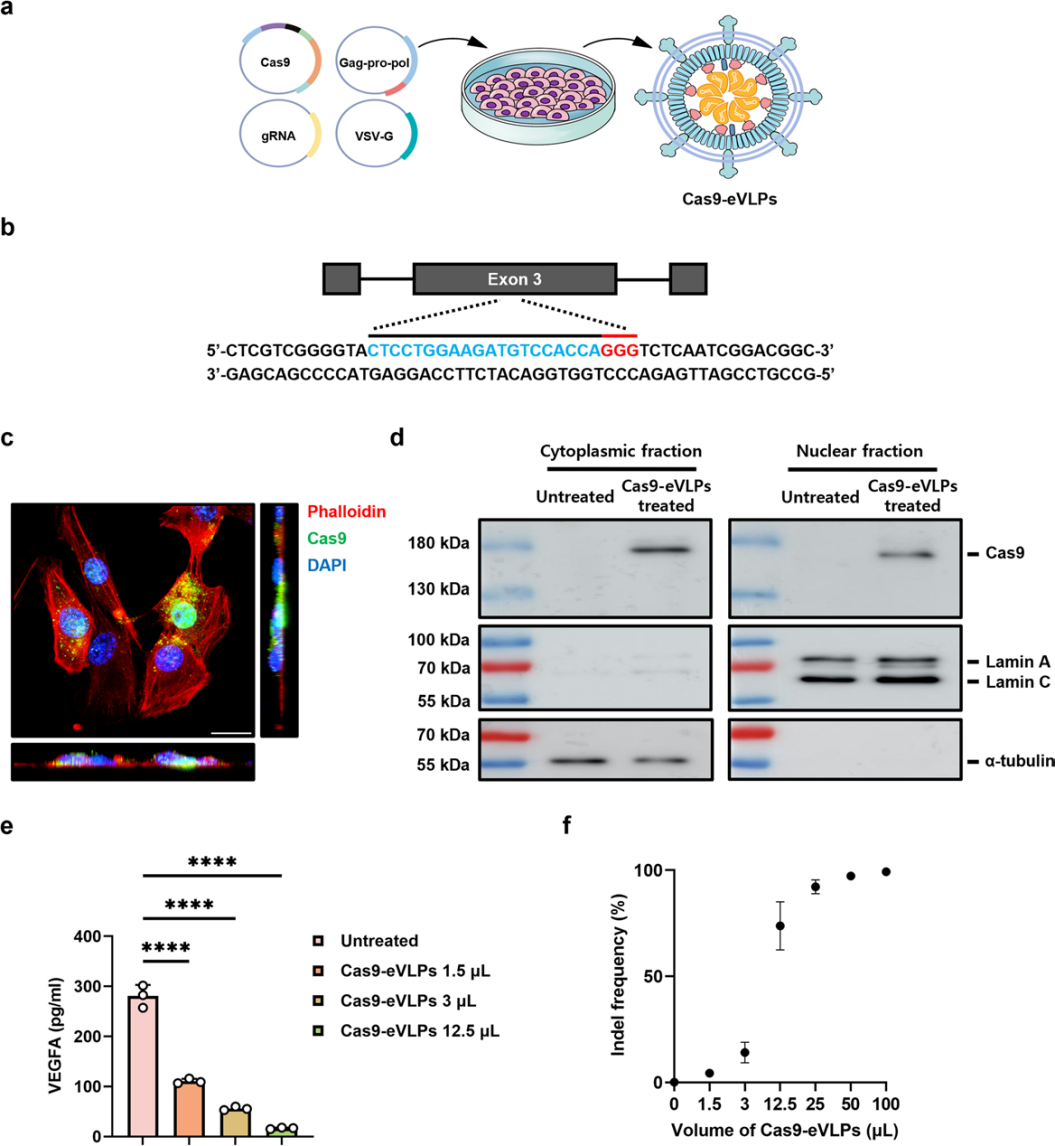
Cas9-eVLPs effectively suppress *Vegfa* expression in vitro. **a** Packing system and the process for producing Cas9-eVLPs. **b** Target sequence in the *Vegfa* locus. The PAM sequence and the sgRNA target sequence are shown in red and blue, respectively. **c** Immunostaining of Cas9 in transduced NIH-3T3 cells 24-h post-transduction. DAPI (Blue), Cas9 (Green), Phalloidin (Red). Scale bar: 20 μm. **d** Nuclear and cytoplasmic fractions were extracted from NIH/3T3 cells. Western blotting analysis of Cas9 in cytoplasmic and nuclear fractions after treatment with or without Cas9-eVLPs. Lamin A/C and α-tubulin were used as nuclear and cytoplasmic markers, respectively. **e** VEGFA concentration in the culture supernatant of NIH/3T3 cells. Enzyme-linked immunosorbent assay of fresh culture media collected 3 days after Cas9-eVLPs transduction. Each microliter of solution contained 4.3 × 10^10^ Cas9-eVLPs (*n* = 5). **f** Indel frequencies at the target site within the *VEGFA* gene in HEK293T cells. Each microliter of solution contains 4.3 × 10^10^ Cas9-eVLPs (*n* = 3). Data are presented as mean ± SD. Statistical analyses were done using one-way ANOVA followed by Tukey’s post hoc multiple comparison tests. ****p < 0.0001

Initially, Cas9-eVLPs were produced by VSV-G, MMLVgag–pol, MMLVgag–3xNES–Cas9, and *Vegfa* sgRNAs transfected into HEK293T-producer cells (Fig. 1a). To evaluate the efficacy of eVLP-mediated Cas9 RNPs delivery in vitro, we identified the Cas9 target site within the mouse *Vegfa* gene, as described in our previous study (Fig. 1b) [40]. Immunocytochemistry images taken 1 day post-transduction showed abundant Cas9 present within the cells and consistent co-localization in DAPI-positive regions (Fig. 1c). The intracellular presence of Cas9 was further supported based on cytosolic-nuclear fractionation assay. We assessed the purity of the cytoplasmic-nuclear fraction using α-tubulin as a specific marker for the cell cytosol and Lamin A/C served as a marker for the nuclear envelope. The results revealed increased Cas9 levels in the cytoplasm and nucleus, suggesting the potent delivery capacity of eVLPs (Fig. 1d, Additional file 1: Fig. S1a). Next, changes in the VEGFA concentration of the conditioned medium were measured using enzyme-linked immunosorbent assay. In samples collected 3 days after transduction, VEGFA production decreased proportionally with the dose of Cas9-eVLPs (Fig. 1e). Additionally, we confirmed the editing potential of the generated Cas9-eVLPs in vitro. As the target sequence in the *VEGFA* gene is the same between humans and mice, we transduced Cas9-eVLPs into HEK293T cells and performed deep sequencing to analyze the insertion and deletion (indel) frequency at the target site. Consistent with the changes in protein levels, we observed dose-dependent disruption of the *VEGFA* locus in the cells treated with Cas9-eVLPs, reaching a peak indel frequency of approximately 99% in the bulk cell population (Fig. 1f).

### In vivo knockout of Vegfa in RPE through subretinal injection of Cas9-eVLPs

**Fig. 2:**
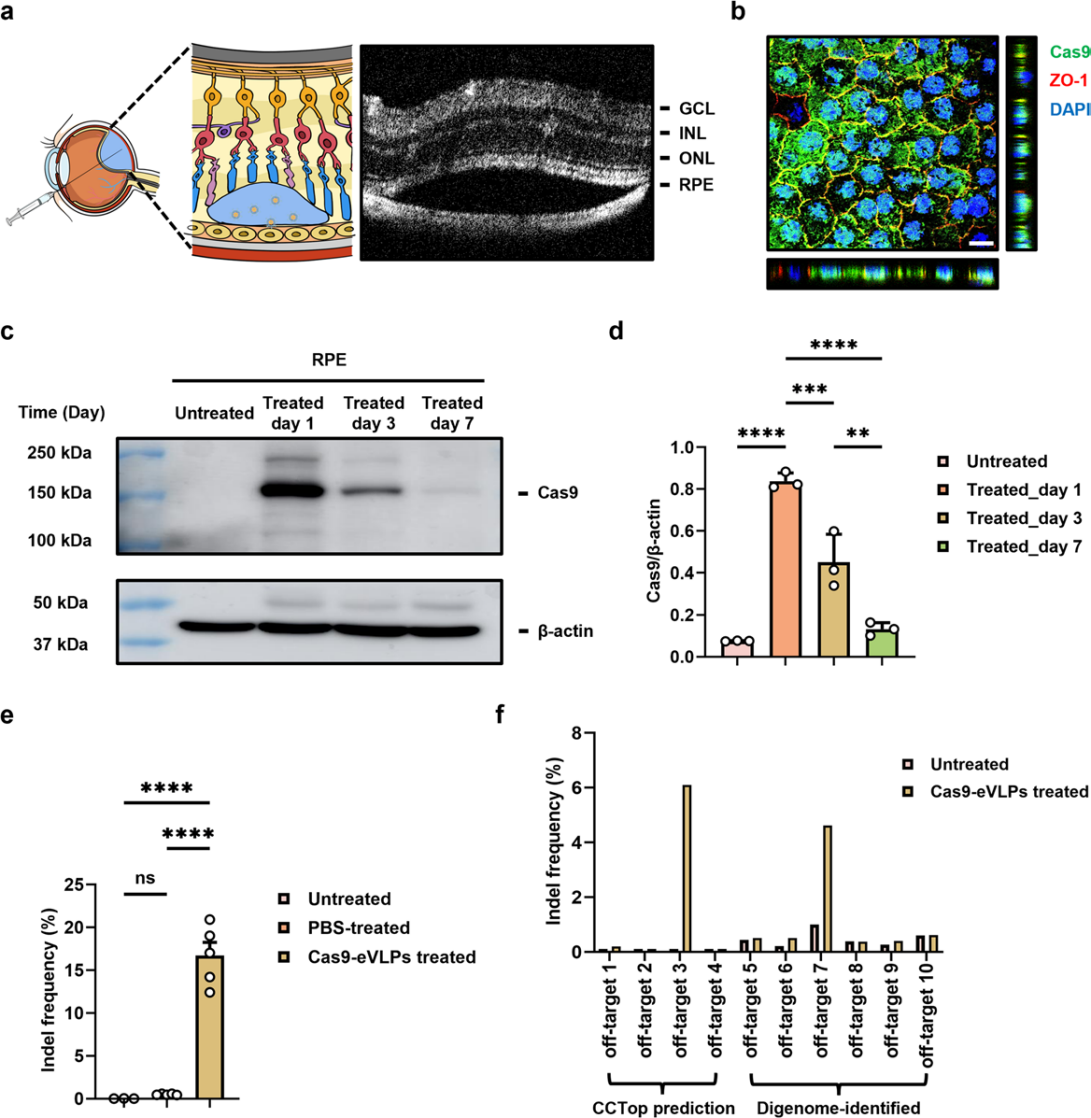
In vivo knockout of *Vegfa* from a subretinal injection of Cas9-eVLPs. **a** Representative OCT images of the mouse retina after subretinal injection of Cas9-eVLPs. *GCL* ganglion cell layer, *INL* inner nuclear layer, *ONL* outer nuclear layer, *RPE* retinal pigment epithelium. **b** Representative image of RCS complexes whole-mount at 1 day post-injection of Cas9-eVLPs into the mouse subretinal space. DAPI (Blue), Cas9 (Green), ZO-1 (Red). Scale bar: 20 μm. **c** Representative Western blotting analysis to measure the level of Cas9 protein in the mouse RPE tissue at 1, 3, and 7 days after Cas9-eVLPs transduction. **d** Histogram showed the densitometric analysis of the levels of Cas9 to β-actin levels (*n* = 3). **e** Following treatment with Cas9-eVLPs, indel frequencies at the *Vegfa* target site were determined in genomic DNA harvested from selected mouse RPE tissue. Samples were collected 1 week after subretinal delivery. *n* = 5 (Cas9-eVLPs-treated group), *n* = 5 (PBS-treated group), or *n* = 3 (untreated group). **f** Indel frequencies at predicted off-target sites of *Vegfa* in pooled RPE tissue dissected from *n* = 5 (Cas9-eVLPs-treated group) or *n* = 3 (untreated group). Off-target 1–4 were predicted using CCTop, and off-target 5–10 were identified by Digenome-seq. Data are presented as mean ± SD. Statistical analyses were done using one-way ANOVA followed by Tukey’s post hoc multiple comparison tests. ns, not significant. **p < 0.01, ***p < 0.001, ****p < 0.0001

To determine the efficacy of Cas9-eVLPs in the mouse RPE, we initially traced the spread after injection. As the blood-retinal barrier limits the distribution of systemically administered compounds, Cas9-eVLPs were delivered into the subretinal space via subretinal injection. Following verification, the highest transduction efficiency in mouse RPE was achieved with a 2 μL cargo volume, consequently establishing 4.3 × 10^10^ Cas9-eVLPs/2 μL as the delivery concentration (Additional file 1: Fig. S2a, b). Optical coherence tomography (OCT) confirmed the successful delivery of Cas9-eVLPs into the subretinal space, which induced artifactual retinal detachment (Fig. 2a). Notably, immunofluorescence positive signals for the Cas9 protein were observed within the nuclei of the RPE tissue 1 day post-injection (Fig. 2b). Western blotting analysis was performed to further investigate the temporal degradation of Cas9 protein in vivo. Cas9 was detected in the RPE at 1 and 3 days post-injection but was nearly undetectable at 7 days post-injection, indicating a transient presence of Cas9 protein in the RPE (Fig. 2c, d, Additional file 1: Fig. S1b). To assess the in vivo gene-editing potential of Cas9-eVLPs, we first performed an initial analysis using genomic DNA extracted from the entire RPE tissue. This revealed an average indel frequency of 6.4% at the target site (Additional file 1: Fig. S3). To better understand the distribution of Cas9, we examined Cas9 protein distribution within the RPE/choroid/sclera (RCS) complex using low-magnification whole-mount imaging. Interestingly, Cas9 localization was not uniform throughout the tissue but instead restricted to distinct regions, with an average coverage of only 27.2% of the RPE (Additional file 1: Fig. S2). This focal distribution likely reflects the localized uptake of Cas9-eVLPs at the injection site and suggests that whole-RPE analysis may underestimate editing efficiency. To more accurately quantify on-target editing, we refined our methodology by isolating genomic DNA specifically from the RPE region corresponding to the injection site. This area was identified via indirect ophthalmoscopy. Targeted deep sequencing of genomic DNA from these selected regions revealed a significantly higher average indel frequency of 16.7%, with a maximum of 20.9%, thereby confirming efficient Cas9-mediated editing in RPE cells within the exposed tissue areas (Fig. 2e). Notably, no editing was detected in retinal cells, confirming the precision of genome editing in the targeted RPE regions (Additional file 1: Fig. S4). To evaluate off-target activity, we conducted deep sequencing of the top four off-target sites predicted by the CCTop prediction tool [41]. No significant off-target editing was observed, except at off-target site 3, located in an intron of the *Acad12* gene, where a 6.1% indel frequency was detected (Fig. 2f). In addition, we performed a genome-wide off-target analysis using Digenome-seq. Deep sequencing of off-target sites identified by Digenome-seq revealed that only off-target site 7, located within an intron of the *Tmem104* gene, showed approximately 3.6% indels detected in Cas9-eVLP-treated samples after subtracting the 1% background indels observed in untreated RPE (Fig. 2f). Next, in an effort to improve on-target editing efficiency, we increased the eVLP injection dose to the maximum concentration achievable by our production method—a fivefold increase (2.15 × 10^11^ particles/eye). Surprisingly, histological analysis revealed that this higher dosage induced severe retinal degeneration in the injected area and was associated with a marked reduction in indel frequency (Additional file 1: Fig. S5a–c), potentially due to VSVG-mediated toxicity [42, 43].

### Vegfa-targeting Cas9-eVLPs mitigate CNV formation in a mouse model of wet AMD

**Fig. 3:**
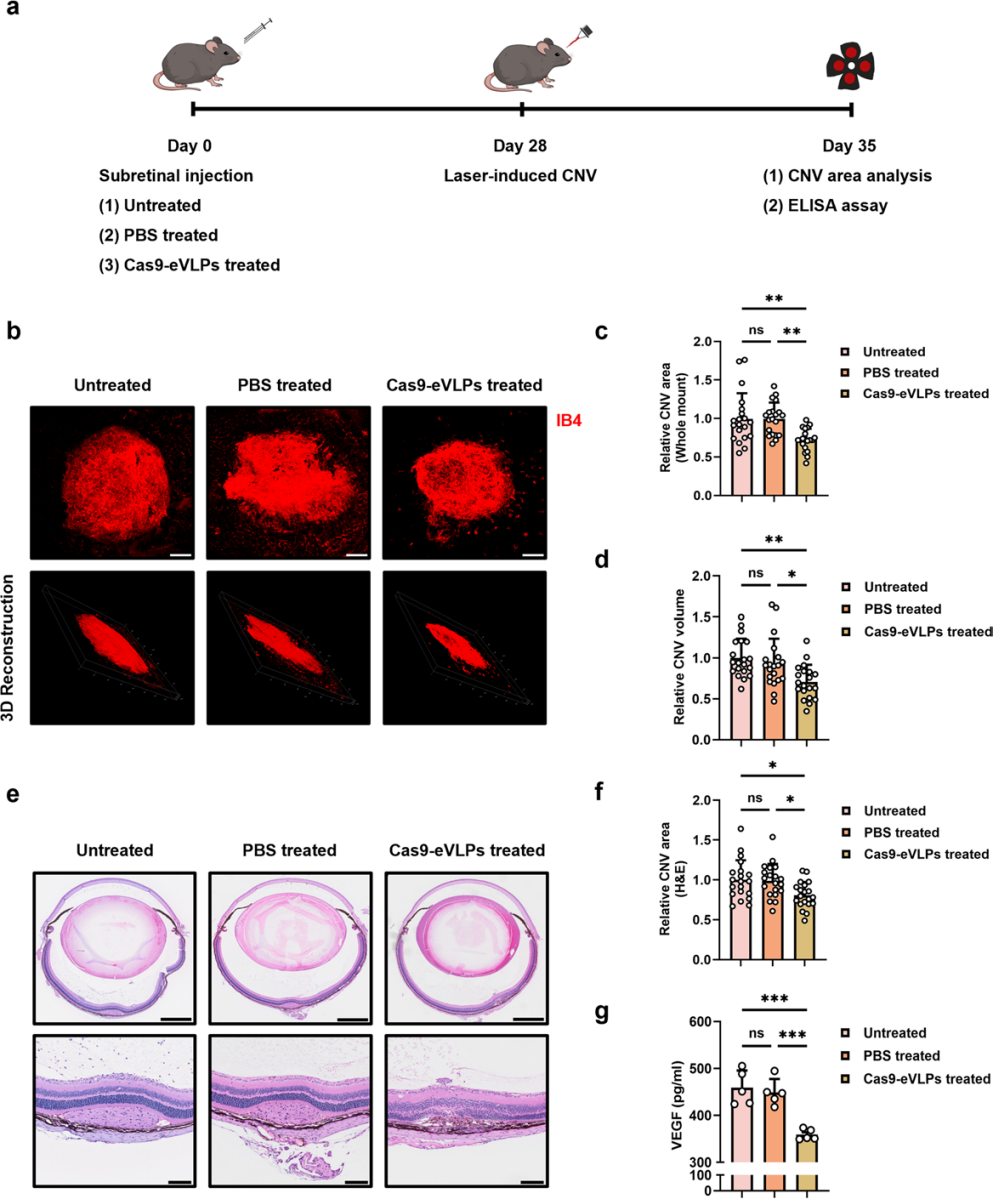
Effect of Cas9-eVLPs targeting *Vegfa* against CNV lesion formation. **a** Experimental workflow of LI-CNV mouse model induced 4 weeks after subretinal injection. Seven days following laser treatment, analysis of the CNV was performed to evaluate the extent of neovascularization and changes in VEGFA protein levels in RPE tissue. **b** Representative laser-induced CNV areas in IB4-stained RCS complexes flat mounts of mouse eyes from untreated, PBS-treated, or Cas9-eVLPs-treated mice. Scale bar: 100 μm. Representative images of confocal microscopy generated 3D side views of CNV lesions in RCS complexes flat mounts from untreated, PBS-treated, or Cas9-eVLPs-treated mice. **c** Quantification of CNV area from untreated, PBS-treated, or Cas9-eVLPs-treated mice (*n* = 20). **d** Quantification of CNV volume from untreated, PBS-treated, or Cas9-eVLPs-treated mice (*n* = 20). **e** H&E staining of retinal section image from untreated, PBS-treated, or Cas9-eVLPs-treated mice at day 7 post laser operation. CNV lesion with collagen visible and infiltrated cells in the sclera and within the lesion. Scale bars: 500 μm (top), 100 μm (bottom). **f** Quantification of CNV lesion area from the H&E images from untreated, PBS-treated, or Cas9-eVLPs-treated mice (*n* = 20). **g** Quantification of VEGFA protein levels from RPE tissue of untreated, PBS-treated, or Cas9-eVLPs-treated mice using ELISA assay (*n* = 5). Data are presented as mean ± SD. Statistical analyses were done using one-way ANOVA followed by Tukey’s post hoc multiple comparison tests. ns, not significant. *p < 0.05, ** p < 0.01, ***p < 0.001

Encouraged by the high frequency of *Vegfa* disruption by Cas9-eVLPs in vitro and in vivo, we investigated their therapeutic efficacy in choroidal neovascularization using a mouse model of wet AMD. Immediate laser surgery could not be performed due to retinal stretching induced by fluid accumulation in the subretinal space post-injection. A 4-week recovery period was required before laser surgery could be performed (Additional file 1: Fig. S6). Therefore, the LI-CNV mouse model was established after structural recovery from iatrogenic retinal detachment (Fig. 3a). Seven days after the laser operation, therapeutic efficacy was assessed by measuring the CNV area and volume. Compared to the untreated and PBS-treated groups, the group that received *Vegfa*-targeting Cas9-eVLPs treatment showed effectively decreased CNV formation (Fig. 3b–d). Histological hematoxylin and eosin (H&E) staining revealed scar-like choroidal tissue beneath the laser retinal site and neovascularization invading the outer retina, both of which were notably diminished in the Cas9-eVLPs-treated mice (Fig. 3e, f). Subsequent measurement of VEGFA protein levels in RPE tissue, known to play a pivotal role in CNV occurrence, demonstrated a significant reduction in Cas9-eVLPs-treated mice compared to the untreated and PBS-treated groups (Fig. 3g).

### Vegfa-targeting Cas9-eVLPs did not induce retinal anatomic and functional toxicity

**Fig. 4:**
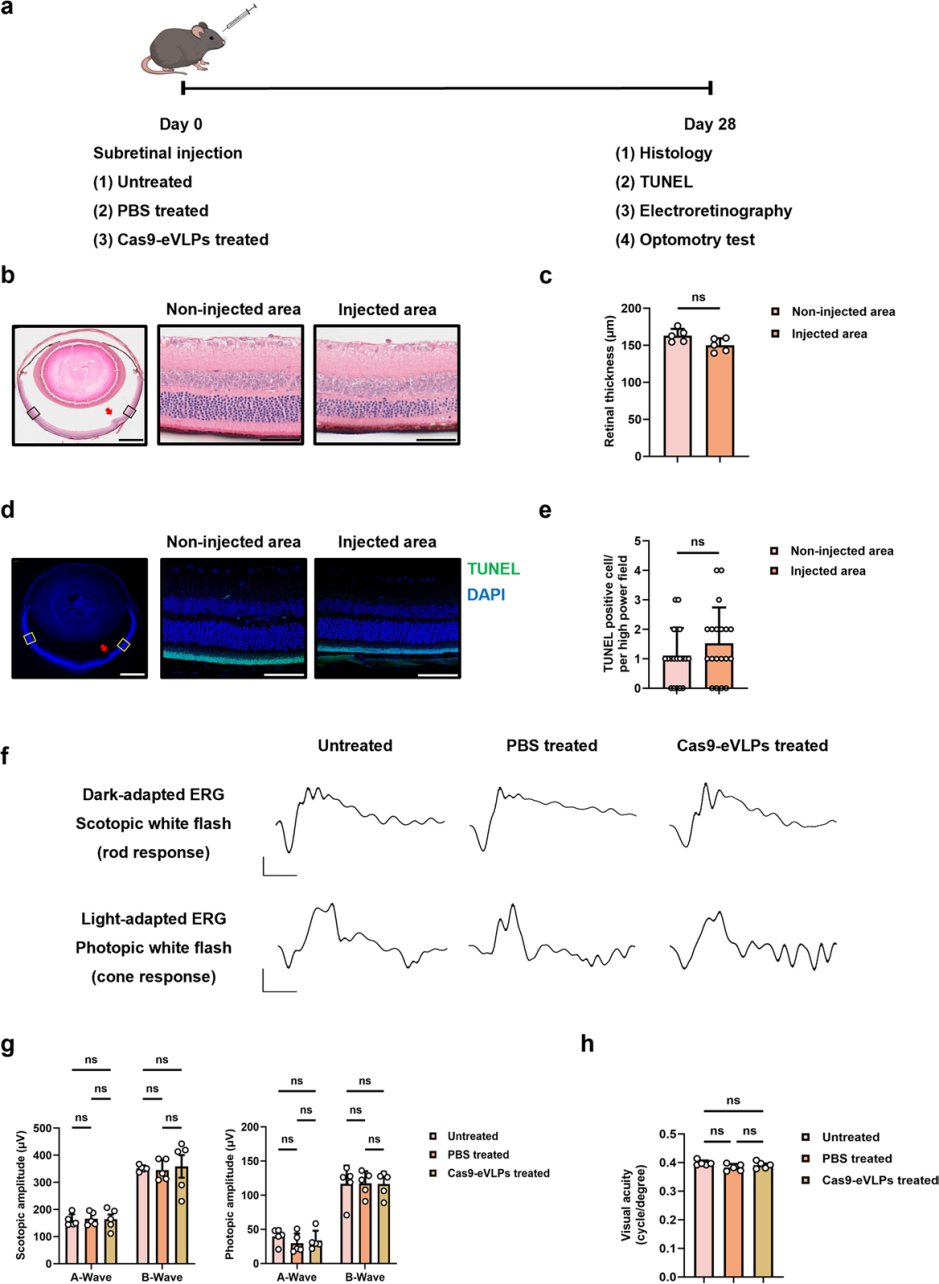
Cas9-eVLPs treatment did not induce anatomical or functional toxicity in the retina. **a** Experimental workflow of retinal anatomical and functional test. **b** Representative H&E staining images after 4 weeks of subretinal injection. Red arrows indicate subretinal injection site. The area within the black box was enlarged and is shown to the right. Scale bars: 500 μm (right), 50 μm (middle), 50 μm (left). **c** Quantification of retinal thickness in proximity to the subretinal injection site and mirroring locations in the contralateral hemisphere of the eyeball (*n* = 5). **d** Representative micrographs of retinal sections evaluated for apoptosis using the TUNEL assay. Red arrows indicate subretinal injection site. The area with the yellow box was enlarged and is shown to the right. Scale bars: 500 μm (right), 50 μm (middle), 50 μm (left). **e** Quantification of TUNEL-positive cells in proximity to the subretinal injection site and mirroring locations in the contralateral hemisphere of the eyeball (*n* = 20). **f** Representative scotopic and photopic ERG waveform from untreated, PBS-treated, or Cas9-eVLPs-treated mice. Scale bars: 30 ms (x-axis), 100 μV (y-axis, top), 50 μV (y-axis, bottom). **g** Amplitudes of a- and b-waves of scotopic and photopic responses (*n* = 5). **h** Optomotor response was quantified in untreated, PBS-treated, or Cas9-eVLPs-treated mice (*n* = 5). Data are presented as mean ± SD. Statistical analyses were done using Student’s t test or one-way ANOVA followed by Tukey’s post hoc multiple comparison tests. *ns* not significant

To evaluate the potential retinal histopathological and morphometric changes following Cas9-eVLPs administration, Cas9-eVLPs were administered to 8-week-old mice via subretinal injection (Fig. 4a). Following a 4-week recovery period, histological examinations with H&E staining were performed to assess retinal structure. Retinal thickness was determined by measuring the distance between the inner limiting membrane and the RPE layer. Although subretinal injection is an invasive delivery method that inevitably leaves scars on the retina, no discernible changes in retinal thickness were observed when comparing the Cas9-eVLPs-injected areas with non-injected areas (Fig. 4b, c). To further validate this result, age-matched normal mice were used as untreated and PBS-treated groups for comparison with the Cas9-eVLPs-injected mice. We confirmed that the samples were sectioned on the same plane by measuring the X-axis length of the lens, and founded that no difference between the three groups (Additional file 1: Fig. S7a–c). Subsequently, a TUNEL assay was conducted to assess apoptosis extent revealed no significant differences in the number of apoptotic cells, as well as compared to that in the untreated and PBS-treated groups (Fig. 4d, e, Additional file 1: Fig. S8a, b). To evaluate potential retinal functional changes, full-field electroretinography (ffERG) recording and OptoMotry response tests were performed 4 weeks after Cas9-eVLPs administration. Dark-adapted ERG recordings were obtained after a dark adaptation period of over 16 h. Following the completion of dark-adapted ERG recordings, the mice were exposed to light conditions for at least 15 min to maximize the cone cell response, and light-adapted ERG recordings were obtained. A comparison of scotopic and photopic responses between Cas9-eVLPs-treated, untreated mice, and PBS-treated mice revealed no significant reduction (Fig. 4f, g). Additionally, the spatial thresholds for virtual rotating stimuli did not decrease in the Cas9-eVLPs-treated mice (Fig. 4h). Moreover, retinal microglia in the Cas9-eVLPs group were not significantly activated compared with those in the untreated and PBS-treated groups (Additional file 1: Fig. S9a, b).

## Discussion

Herein, we report a system that allows for Cas9 RNPs delivery through eVLPs for efficient “hit-and-run” genome editing without transferring viral vector genetic material. Using eVLPs to assemble Cas9 RNP complexes, we successfully demonstrated potent *Vegfa* knockout both in vitro and in vivo, showing reductions in the CNV formation in a mouse model of wet AMD. Crucially, our method produced indel frequencies in mouse RPE that were comparable to those reported previously [23, 40, 44, 45]. These results strongly support eVLPs as a delivery platform for genome editing to help treat retinal diseases.

Advancements in genome sequencing have improved its accuracy, speed, and cost-effectiveness, establishing genome editing as a cornerstone of precision medicine, which aims to maximize treatment efficacy while minimizing risks by tailoring interventions to the individual characteristics of each patient [46, 47]. To achieve tailored interventions through gene editing, the accurate delivery of CRISPR-based payloads to target organs or cells is essential. However, despite some progress, effective macromolecule delivery to the intended target tissue without off-target effects or unwanted immune responses remains challenging. Selecting an appropriate delivery system to facilitate the transport of gene-editing cargo to target tissues is a critical step in overcoming the remaining hurdles.

VLPs are non-replicating particles that are structurally similar to native viruses but lack viral genetic material. They are typically produced by expressing viral structural proteins, such as Gag, in host cells, where they assemble into nanoparticles that can encapsulate mRNA or RNP cargo. Because VLPs do not carry viral genomes, they cannot replicate, making them a safer alternative to viral vectors for therapeutic delivery. In particular, macromolecular gene editing cargo can be fused to the Gag polyprotein and encapsulated in pseudotyped VLPs, leading to transient genome editing [48, 49]. Compared to conventional AAV- and LNP-mediated genome editing strategies, eVLPs offer significant advantages as a virus-free alternative. They allow tissue targeting, prevent genomic integration, avoid long-term expression of the Cas9, and promote relatively high editing efficiencies for precise and tailored delivery [36, 48, 50].

Recently, eVLP for efficient delivery of prime editor RNPs in vivo was reported to achieve significant improvements in editing efficiency and demonstrate therapeutic potential in mouse models of genetic blindness [51]. Another study introduced the RIDE system, a customizable VLP for delivering CRISPR-Cas9 RNPs, which enabled efficient and cell-specific gene editing in a mouse model of ocular neovascularization and non-human primate model of Huntington’s disease [52]. Structurally, RIDE and eVLP differ in their cargo incorporation strategies. RIDE uses MS2 stem-loop-mediated packaging for programmable cell targeting, whereas eVLPs employ optimized Gag–cargo fusion and linker designs to enhance protein loading and release. Functionally, RIDE enables cell-type-specific delivery for precise gene editing, whereas eVLPs offer broad tissue accessibility and transient expression [37, 52]. These findings collectively indicate that adapting the VLP platform evolution approach may be broadly useful for developing delivery vehicles that overcome existing limitations.

In this study, we initially demonstrated the potent editing capabilities of Cas9-eVLPs in cellular environments. As the dose of eVLPs increased, the indel frequencies concomitantly led to a reduction in VEGFA protein levels (Fig. 1). To evaluate in vivo performance, we used subretinal injections to deliver genome-editing payloads. However, analyzing the whole RPE resulted in initial indel frequencies that were lower than those reported previously [23, 40]. Immunofluorescence analysis revealed that the cargo delivered into the subretinal space was predominantly localized within the injected area (Additional file 1: Fig. S2), which is consistent with prior reports [40, 53, 54]. Notably, the size of the transduced area differed among mice, which was most likely due to variations in the injection site and angle [55, 56]. Next, analyzing genomic DNA from the selected RPE tissues showed that indels reached an average of 16.7%, with a maximum of 20.9% (Fig. 2). Subretinal injections require highly precise micro-procedures; however, they are inherently prone to variability between administrations. This variability primarily arises from uneven dispersion of the injected solution and occasional backflow into the vitreous body [55]. As a result, variations in indel frequency were anticipated. Although the off-target site OT3 showed a 6.1% indels, it is located within an intronic region. Encouraged by the in vitro editing efficiency, we increased the eVLP dose in an attempt to achieve higher indel frequencies at the target site, aiming to elicit a more pronounced phenotypic effect. However, this approach inadvertently led to retinal degeneration and failed to improve the indel frequency (Additional file 1: Fig. S5). High-dose or prolonged VSV-G expression causes high levels of cytotoxicity, including cell fusion and cell death. Moreover, direct delivery of Cas9 RNP enables genome editing in the retina but shows dose-dependent toxicity [42, 43, 57]. These findings indicate that optimizing the delivery concentration of gene-editing enzymes is a prerequisite for achieving therapeutic effects. Additionally, long-term suppression of VEGF is reportedly linked to changes in retinal tissues [58]. However, our results showed that appropriate doses of Cas9-eVLPs resulted in a notable absence of significant alterations in retinal anatomy or functionality (Fig. 4). Although the retinal thickness within the injected area was relatively thin, this could be attributed to the invasive nature of the subretinal space delivery route. Subsequent analyses confirmed that the subretinal injection of Cas9-eVLPs *Vegfa*-targeting effectively inhibited neovascularization in the LI-CNV mouse model (Fig. 3). Taken together, our results demonstrate the sustained suppression of VEGFA in a mouse model of wet AMD, with the benefits significantly outweighing any potential risks associated with the therapeutic approach. However, the indels in the RPE were slightly behind the strong delivery capacity of eVLPs. We believe that the inherent variability of subretinal injections requires a well-trained operator for optimal execution. Furthermore, the precise selection of successfully transduced RPE cells helps to obtain realistic editing results. Finally, determining the optimal concentrations for injections necessitates rigorous evaluation.

Current research data indicate that AAV-based gene therapies demonstrate a well-established safety profile, high biocompatibility, and efficient delivery of therapeutic payloads to diverse relevant tissues [12, 59]. AAV-based vectors have been utilized in over 200 active clinical trials, and drugs have been approved by the US Food and Drug Administration, demonstrating favorable safety outcomes and meaningful therapeutic benefits across a range of genetic disorders [60, 61]. Given the encouraging data from these studies, AAV-based gene therapy is emerging as an effective therapeutic strategy for correcting conditions with unmet medical needs. Nonetheless, the current efficiency of these tools is not sufficient for therapeutic use. Although a non-pathogenic vector can be applied to an immune-privileged site, such as the retina, AAV-mediated retinal gene therapy is still recognized by the immune system and elicits immune responses [25]. These responses can cause ocular inflammation, referred to as gene therapy-associated uveitis, and trigger the production of neutralizing antibodies that may reduce the efficiency of subsequent vector administrations, such as when treating the contralateral eye [62–64]. Hence, there is a global effort to develop non-viral vectors aimed at delivering large cargo while eliminating viral components. In comparison to our previous studies, the development of the eVLP delivery system has been successful, particularly regarding indel efficiency, which was comparable to the results of AAV delivery in the same animal model [44]. Moving forward, studies should increasingly experiment with using eVLP as an alternative platform.

## Conclusions

In conclusion, eVLP-mediated delivery of Cas9 RNPs enables efficient and targeted gene editing in the mouse RPE via subretinal injection while minimizing unintended editing in the retina. Moreover, *Vegfa*-targeting Cas9-eVLPs effectively attenuated choroidal neovascularization, underscoring the potential of this approach as a therapeutic strategy for wet AMD. These findings also highlight the importance of optimizing delivery concentration to achieve a balance between efficacy and safety. Taken together, our results support the potential of eVLPs as a delivery platform for the treatment of retinal diseases.

## Methods

### Cell culture

NIH/3T3 (CRL-1658) and HEK293T (CRL-3216) cells were obtained from ATCC. After thawing, the cells were verified to be mycoplasma negative before further culture. Cells were cultivated in Dulbecco’s modified Eagle’s medium (LM007-01; Welgene). The media contained 10% fetal bovine serum (16000044; Thermo Fisher Scientific) and 100 units/mL penicillin/streptomycin (15140–122; Thermo Fisher Scientific). The cells were maintained in a 5% CO_2_ incubator at 37 °C.

### Construction of plasmid vectors

To construct *Vegfa* sgRNA plasmid, an sgRNA-expressing vector (104174; Addgene) was digested with BsaI. sgRNA oligomers were annealed, phosphorylated with T4 PNK, and Ligated with the Linearized vector. The oligomer sequences are shown in Additional file 2: Table S1. VSV-G plasmid (8454), MMLVgag-pol plasmid (35614), and MMLVgag-3xNES-Cas9 plasmid (181752) were obtained from Addgene.

### eVLP production and purification

To produce Cas9-eVLPs, 1 × 10^7^ HEK293T cells were seeded on 150-mm cell culture dishes containing DMEM. After incubation for 16 h, DMEM was exchanged with fresh medium and a mixture of plasmids expressing VSV-G (400 ng), MMLVgag–pol (3375 ng), MMLVgag–3xNES–Cas9 (1125 ng), and an *Vegfa* sgRNA (4400 ng) were co-transfected per dish using polyethyleneimine. At 48 h after transduction, cell supernatant was harvested and centrifuged for 5 min at 500 g to remove cell debris. The clarified supernatant was filtered through a Millex-HV 0.45-μm low protein-binding membrane (Millipore). For Cas9-eVLPs that were used in cell culture, the filtered supernatant was concentrated 100-fold using PEG-it Virus Precipitation Solution (LV825A-1; System Biosciences) according to the manufacturer’s protocols. For Cas9-eVLPs that were injected into mice, the filtered supernatant was concentrated 2000-fold via ultracentrifugation using a cushion of 20% (w/v) sucrose in PBS. Ultracentrifugation was performed at 26,000 rpm for 2 h (4 °C) using an SW28 rotor in an Optima XPN Ultracentrifuge (Beckman Coulter). Following ultracentrifugation, eVLP pellets were resuspended in cold PBS (pH 7.4) and centrifuged at 1000 g for 10 min to remove debris. eVLPs were frozen at a rate of − 1 °C/min and stored at − 80 °C.

### Quantification of eVLP particles

The concentration of eVLPs (number of VLP particles per microliter) was measured by quantifying MuLV p30 protein with the MuLV Core Antigen ELISA kit (Cell Biolabs; VPK-156) following the manufacturer’s protocol. Recombinant MuLV p30 standard included in the kit was used to generate the standard curve and the concentration of VLP-associated p30 protein was calculated with the assumption that 20% of the observed p30 in solution was associated with VLPs, as was previously reported [65].

### Deep sequencing of target genomic loci

Genomic DNA was extracted either from cultured cells using the Wizard Genomic DNA Purification Kit (A2920; Promega) or from mouse RPE using the DNeasy Blood & Tissue Kit (69504; QIAGEN). Target sequences were PCR-amplified using Phusion® High-Fidelity DNA Polymerase (M0530S; NEB). The PCR primers used for targeted deep sequencing are listed in Additional File 2: Table S1. To evaluate indel frequencies in HEK293T cells, an initial PCR was performed using 200 ng of genomic DNA and primers containing Illumina adaptor sequences. Then, 2 µL of a 1:10 dilution of the PCR amplicon was subjected to a second PCR using primers with barcode sequences. After gel purification with the MEGAquick-spin Total Fragment DNA Purification Kit (17290; iNtRON Biotechnology), the amplicons were analyzed using the MiniSeq platform (Illumina). For evaluation of indel frequencies in mouse RPE, PCR was performed using 50 ng of genomic DNA, following the same protocol described above. After gel purification, the resulting amplicons were sequenced using both the MiniSeq and MiSeq platforms (Illumina).

### Analysis of off-target editing

The top-four predicted off-target sites for *Vegfa* sgRNA were identified using the CCTop-CRISPR/Cas9 target online predictor [41]. Predicted off-target sites were amplified using 2 × Taq PCR smart mix (STD01-M50h; Solgent). An initial PCR was performed to amplify the target and potential off-target sites in a 30-µL reaction volume containing 30 ng of genomic DNA from the RPE. To attach Illumina index sequences, a second round of PCR was performed using 1 µL of the initial PCR product in a 30-µL reaction volume. The purified PCR products were pooled at equal molar ratios for sequencing on the MiniSeq platform (Illumina). Cas-analyzer was used to analyze indel frequencies at the off-target sites [66]. The sequences of the off-target sites and the primers used for deep sequencing are listed in Additional file 2: Table S2.

### Digenome-seq

We conducted Digenome-seq as previously described [67, 68]. Genomic DNA was extracted using the DNeasy Tissue Kit (QIAGEN) following the manufacturer’s protocol. In brief, recombinant Cas9 nuclease (100 nM) was pre-incubated with *Vegfa* sgRNA at room temperature for 10 min to generate Cas9-sgRNA complex. Next, the complex was mixed with 8 µg of mouse genomic DNA in a reaction buffer (100 mM NaCl, 50 mM Tris–HCl, 10 mM MgCl_2_, 100 µg mL^−1^ bovine serum albumin, at pH 7.9) and incubated for 8 h at 37 °C. Following digestion, the DNA was treated with RNase A and proteinase K, and then re-purified using the DNeasy Tissue Kit (QIAGEN). The digested DNA was subsequently fragmented using the Covaris system. The resulting DNA fragments were blunt-ended using End Repair Mix (illumina) and then ligated with sequencing adaptors for library preparation. Whole-genome sequencing (WGS) was performed on the Illumina HiSeq X Ten platform at a sequencing depth of 30 × . DNA cleavage scores were calculated with previously used source codes (https://github.com/chizksh/digenome-toolkit2) [67]. All sites captured by Digenome-seq are shown in Additional file 2: Table S3.

### Immunocytochemistry

NIH/3T3 cells were seeded into 8-well culture slide (30508; SPL Life Sciences) at 3 × 10^4^ cells per well. One day after Cas9-eVLPs transduction, cells were fixed in 4% paraformaldehyde (PFA, P2031; Biosesang) for 15 min at room temperature and stained with the Cas9 mouse monoclonal antibody (1:200, 14697S; Cell Signaling Technology) overnight at 4 °C. On the following day, the cells were rinsed three times with PBS, then incubated in Alexa Fluor 488 Goat anti-Mouse IgG (H + L) Highly Cross-Adsorbed (1:250, A32723; Invitrogen) at room temperature for 2 h. After rinsed with PBS three times, cells were counterstained with Phalloidin Labeling Probes (1:400, A12381; Invitrogen) and DAPI (1:1000, D9542; Sigma-Aldrich) at room temperature, respectively. After rinsing three times, the culture slide was mounted with a sufficient amount of mounting solution. The imaging was performed using confocal microscopy (Leica Microsystems).

### Western blotting

In vitro, NIH/3T3 cells were seeded into a 60-mm culture dish (20060; SPL Life Sciences) at 3 × 10^5^ cells per well. After Cas9-eVLPs transduction, cells were fractionated using Nuclear and Cytoplasmic Extraction Reagents (78833; ThermoFisher) according to the manufacturer’s protocols. In vivo, the eye was harvested at 1, 3, and 7 days after Cas9-eVLPs treatment, and protein was extracted from the RPE tissue using a RIPA buffer (RC2002-050; Biosesang) containing protease inhibitor (p3100-001; GenDEPOT). The protein concentration of each sample was determined using the BCA Protein Quantitation Kit (23228; ThermoFisher). Protein samples were then separated via SDS-PAGE and transferred to polyvinylidene difluoride filter membranes. The membranes were blocked with 5% skim milk in TBST (Tris buffered saline containing 0.1% Tween) at room temperature for 1 h, and then incubated with primary antibodies overnight at 4 °C. The following primary antibodies were used: Lamin A/C (1:1000, 4777S; Cell Signaling Technology), α-Tubulin (1:1000, sc-23950; Santa Cruz Biotechnology), β-actin (1:10000, A1978; Sigma-Aldrich), and Cas9 (1:1000, 14697S; Cell Signaling Technology). On the following day, membranes were rinsed with TBST 3 times and incubated with horse anti-mouse IgG antibody (1:5000, 7076; Cell Signaling Technology) for 1 h at room temperature. The blots were treated with enhanced chemiluminescence reagent (34095; ThermoFisher) and detect bands using Chemi image system2 (GE Life Sciences). Gel band quantification using ImageJ software, following the steps, converting the gel image to 8-bit grayscale, inverting the image so that bands appear as peaks, and using the gel analysis tool to select regions of interest, according to the protein size (Cas9, 162 kDa; β-actin, 42 kDa). After plotting the lanes, the area under each peak (representing band intensity) is measured.

### Enzyme-linked immunosorbent assay

According to the manufacturer’s protocols, the Quantikine murine VEGF ELISA kit (MMV00; R&D Systems) was used to detect the levels of VEGFA released from NIH/3T3 cells and RPE tissues after Cas9-eVLPs transduction. In brief, collected sample, control, and standard sample were added to each well and mixed by gently tapping the plate frame for 1 min. Thereafter, wells were covered with an adhesive strip and incubate for 2 h at room temperature. Each well was aspirated and washed with Wash Buffer using an autowasher. Thereafter, 100 μL of Mouse VEGF Conjugate was added to each well, which was then covered with a new adhesive strip to incubate for 2 h at room temperature. The aspiration and wash process was then repeated, 100 μL of Substrate Solution were added to each well and incubate for 30 min without light stimulation. Finally, we added 100 μL of Stop Solution and determined the optical density using microplate reader (ELR08IFL; AID Reader Systems).

### Animals

The C57BL/6 (000664) mice were purchased from the Jackson Laboratory. Eight-week-old male mice were employed in all experiments. All animal experiments in this study were approved by the Seoul National University Animal Care and Use Committee (Permit Number: SNU-230731–4-4) and conducted in strict accordance with the guidelines of the Association for Research in Vision and Ophthalmology Statement. Mice were kept under cyclic light (12-on/12-off) with ad libitum access to food and water in approved cages.

### Subretinal injection

Mice were anesthetized with an intraperitoneal injection of tiletamine (25 mg/mL)/zolazepam (25 mg/mL) mixture. After anesthesia, mouse eyes were placed in the proper position, and pupils were dilated with an eye drop containing phenylephrine hydrochloride (5 mg/mL) and tropicamide (5 mg/mL). After opening the eyelid and protruding the eye to expose the equator for convenient injection, a small hole was punctured at the slight posterior of the limbus using a sterile 30-gauge needle. The 33-gauge blunt needle of a microliter syringe was placed through the pre-punctured hole. The needle was then inserted into the subretinal space until the point when mild resistance was felt. The solution was injected slowly with low pressure, and the retinal bleb was checked under the indirect ophthalmoscopy. Mice received a titer of 4.3 × 10^10^ or 2.15 × 10^11^ Cas9-eVLPs into the subretinal space. Moreover, age-matched mice that were not injected served as the untreated group, and mice injected with PBS served as the vehicle group, respectively.

### Laser-induced choroid neovascularization

Eight-week-old male mice were utilized to conduct LI-CNV following treatment with Cas9-eVLPs for 4 weeks. Upon anesthesia, their eyes were dilated using an eye drop solution containing phenylephrine hydrochloride (5 mg/mL) and tropicamide (5 mg/mL). Subsequently, a laser photocoagulator equipped with an indirect headset delivery system (Ilooda) was employed to visualize the retina. The laser parameters were as follows: wavelength: 810 nm; spot size: 200 μm; power: 1 W; exposure time: 100 ms. Adequate laser energy was applied to four locations per eye to induce rupture of Bruch’s membrane. Only burns that resulted in a bubble formation without vitreous hemorrhage were considered for inclusion in the study. On the 7 days post-laser photocoagulation, the eyes were enucleated, fixed in 4% PFA, and prepared for whole-mounted RCS complex formation. Following the immunofluorescence staining procedure with Alexa Fluor 568-conjugated anti-IB4 antibody (1:250, I21413; Invitrogen), the CNV area of all the laser burn sites (four laser burn sites for each eye) was quantitatively analyzed using a built-in measuring tool, the LAS X systems (TCS SP8; Leica Microsystems). CNV volume quantitied using Imaris software (Oxford Instruments).

### Ophthalmoscopy

Prior to checking, phenylephrine hydrochloride/tropicamide mixture were applied topically, and general anesthesia was induced in mice. The Vantage Plus head-worn binocular indirect ophthalmoscope (1205-P-1020; Keeler) with non-contact 78D slit lamp lens (Volk Optical) to retinal general diagnosis while maintaining corneal moisture. After subretinal injection or laser photocoagulation, when complications such as subvitreal hemorrhage were observed by ophthalmoscopy, the mice were discharged from the experiment.

### Immunofluorescence staining

Pups from each group euthanized via CO_2_ inhalation. The ocular globe was enucleated and fixed in 4% PFA for 30 min at room temperature. The cornea and lens were removed, then the retina was dissociated from the RCS complex. The RCS complex was incubated in blocking solution (BP150; Biosolution) at room temperature for 2 h and stained with Cas9 mouse monoclonal antibody (1:200, 14697S; Cell Signaling Technology) overnight at 4 °C. On the following day, we rinsed the stained RCS complex three times and incubated with Alexa Fluor 488 Goat anti-Mouse IgG (H + L) Highly Cross-Adsorbed (1:250, A32723; Invitrogen) at room temperature for 2 h. We then rinsed three times and incubated with an Alexa Fluor 594-conjugated anti-ZO-1 antibody (1:250, 339194; Invitrogen) at room temperature for 2 h. The samples were counterstained with DAPI (1:1000, D9542; Sigma-Aldrich) at room temperature for 15 min. Afterward, the stained RCS complex was placed on a glass slide with the retinal pigment epithelial layer against the glass slide. An adequate amount of mounting solution was added, and a cover slide was placed. For detect activated retinal microglia, the paraffin ribbons followed deparaffinization and heat-induced epitope retrieval procedure. Then, anti-Iba1 antibody (1:50, EPR16588; Abcam) was incubated overnight at 4 °C. After rinsed with PBS 3 times and incubated with Alexa Fluor 488 Goat anti-Rabbit IgG (H + L) Highly Cross-Adsorbed (1:400, A32731; Invitrogen) at room temperature for 2 h. The nuclei were counterstained with DAPI and carry out sealing. Immunostained tissues were observed using a confocal microscope.

### Histology

The mice were euthanized, and the globes were fixed in Hartman’s fixative solution (H0290; Sigma-Aldrich) and 4% PFA for 20 h at room temperature, respectively. After fixation, “windows” were made on the eyeball at the location of the anterior and posterior chambers, as described previously [69]. Briefly, the eye was held in place with a forceps, and a slight incision with a 26-gauge needle was made into the anterior chamber. This step was repeated in the posterior part of the eyeball, which was in line with the window in the anterior chamber. The globes were embedded in paraffin. After 4-μm-thick paraffin sections were prepared, the sections were deparaffinized and hydrated via sequential immersion in graded ethyl alcohol solutions and xylene substitute (6764506; Epredia). H&E staining was performed for histological examination. The retinal thickness from the inner limiting membrane to the retinal pigment epithelial layer was measured using NIS-Elements Imaging Software (Nikon Instruments).

### TUNEL assay

After deparaffinization and hydration of retinal paraffin sections via sequential immersion in graded ethyl alcohol solutions and xylene substitute, the deparaffinized slides were immersed in heated citrate buffer for 10 min. The slides were removed from the hot citrate buffer and cooled in ddH_2_O for 10 min. TUNEL staining was performed using an in situ cell death detection kit (11684809910; Sigma-Aldrich) according to the manufacturer’s protocols, and the nuclei were counterstained with DAPI. Retinal TUNEL-positive cells were counted in randomly selected fields in each group under a confocal microscope.

### Optical coherence tomography

OCT imaging was performed using a custom-built OCT machine developed by the Korea Research Institute of Standards and Science. Mice were anesthetized with an intraperitoneal injection of tiletamine (25 mg/mL)/zolazepam (25 mg/mL) mixture. After anesthesia, mouse eyes were placed in the proper position, and pupils were dilated with an eye drop containing phenylephrine hydrochloride (5 mg/mL) and tropicamide (5 mg/mL). The fully dilated pupil was directed toward the subjective lens. Eyes were kept moisturized with PBS solution during the entire procedure to ensure optimized images. The following OCT system specifications were used, axial resolution: 2.60 µm in tissue (*n* = 1.37), image depth: 1.45 mm in tissue, scan range: 3.0 mm (mouse eye), SLD: 850 nm ± 100 nm and optical power: 0.78 mW.

### Electroretinography

After anesthesia and mydriasis, a recording electrode was placed on the corneal surface, and the reference needle electrode was subcutaneously inserted in the head, with an electrode in the tail serving as the ground. The ffERG was performed using the electrophysiologic system 3000 (UTAS E-3000; LKC Technologies). Mice were adapted in the dark for over 16 h. In the dark-adapted condition, the scotopic responses were recorded using a single dim flash of 0 dB using a notch filter at 60 Hz and a digital bandpass filter ranging from 0.3 to 500 Hz. After recording scotopic responses, the mice were exposed to light for a minimum of 15 min. In the light-adapted condition, photopic responses were recorded in response to a single flash of 0 dB, utilizing the same notch filter and digital bandpass filter settings. The amplitude of the a-wave was measured from the baseline to the lowest negative going voltage, whereas peak b-wave amplitudes were measured from the trough of the a-wave to the highest peak of the positive b-wave. The ERG waveforms were visualized using GraphPad PRISM 9 (GraphPad Software).

### OptoMotry response test

A virtual optomotor system (OptoMotry apparatus; CerebralMechanics) was used to assess visual function. Briefly, the mice were placed on an elevated platform positioned in the middle of an arena created by four inward-facing display monitors. Spatial frequency thresholds were assessed using a video camera to monitor the elicitation of the optokinetic reflex through virtual stimuli projected with sine-wave gratings (100% contrast) on the computer monitors. Experimenters were blinded to the treatment and each animal’s previously recorded thresholds.

### Data analysis

Statistical significance was calculated using GraphPad Prism 9. All data were first assessed for normal distribution, an unpaired t test was used to identify significant differences. Considering the normal distribution and homogeneity of variance data, one-way ANOVA with Tukey’s post hoc tests was used for multiple comparisons. The data sets are presented as mean ± standard deviations (SD). Differences were considered significant at the level of *p* < 0.05.

## Supplementary Information

Additional file 1: Fig. S1. The full gels of Western blotting data in supplementary information. Fig. S2. Cas9-eVLPs delivered subretinally are localized to the injection site. Fig. S3. Indel frequencies from whole RPE tissue. Fig. S4. Indel frequencies from mouse retina. Fig. S5. Excessive delivery of Cas9-eVLPs induces retinal degeneration in mice. Fig. S6. Recovery process of artificially induced retinal detachment after subretinal injection. Fig. S7. Evaluate the toxicity of Cas9-eVLPs by comparing the retinal anatomical structures. Fig. S8. Detection of retinal apoptotic cells by TUNEL assay. Fig. S9. Detection of activation of immune cells in retina.

Additional file 2: Table S1. Oligomers for sgRNA cloning and primers used to amplify the on-target site for NGS. Table S2. Primers used for amplification of off-target sites: Off-targets 1–4 (CCTop-predicted) and Off-targets 5–10 (Digenome-seq-identified with NGG PAM). Table S3. All cleavage sites identified by Digenome-seq and their corresponding DNA cleavage scores.

## Data Availability

The deep sequencing data from this study have been submitted to the NCBI Sequence Read Archive (SRA; https://www.ncbi.nlm.nih.gov/sra/) under accession number PRJNA1117205 [70]. The data analyzed during the current study are available at Zenodo (https://zenodo.org/records/17019773) [71].

## References

[1] Guymer RH, Campbell TG. Age-related macular degeneration. Lancet. 2023;401:1459–72. 36996856 10.1016/S0140-6736(22)02609-5

[2] Wong WL, Su X, Li X, Cheung CM, Klein R, Cheng CY, et al. Global prevalence of age-related macular degeneration and disease burden projection for 2020 and 2040: a systematic review and meta-analysis. Lancet Glob Health. 2014;2:e106-116. 25104651 10.1016/S2214-109X(13)70145-1

[3] Mitchell P, Liew G, Gopinath B, Wong TY. Age-related macular degeneration. Lancet. 2018;392:1147–59. 30303083 10.1016/S0140-6736(18)31550-2

[4] Fleckenstein M, Keenan TDL, Guymer RH, Chakravarthy U, Schmitz-Valckenberg S, Klaver CC, et al. Age-related macular degeneration. Nat Rev Dis Primers. 2021;7:31. 33958600 10.1038/s41572-021-00265-2 PMC12878645

[5] Wong TY, Chakravarthy U, Klein R, Mitchell P, Zlateva G, Buggage R, et al. The natural history and prognosis of neovascular age-related macular degeneration: a systematic review of the literature and meta-analysis. Ophthalmology. 2008;115:116–26. 17675159 10.1016/j.ophtha.2007.03.008

[6] Rosenfeld PJ, Brown DM, Heier JS, Boyer DS, Kaiser PK, Chung CY, et al. Ranibizumab for neovascular age-related macular degeneration. N Engl J Med. 2006;355:1419–31. 17021318 10.1056/NEJMoa054481

[7] Heier JS, Brown DM, Chong V, et al. Intravitreal aflibercept (VEGF trap-eye) in wet age-related macular degeneration. Ophthalmology. 2012;119:2537–48. 23084240 10.1016/j.ophtha.2012.09.006

[8] Dugel PU, Koh A, Ogura Y, Jaffe GJ, Schmidt-Erfurth U, Brown DM, et al. HAWK and HARRIER: phase 3, multicenter, randomized, double-masked trials of brolucizumab for neovascular age-related macular degeneration. Ophthalmology. 2020;127:72–84. 30986442 10.1016/j.ophtha.2019.04.017

[9] Rein DB, Wittenborn JS, Zhang P, Sublett F, Lamuda PA, Lundeen EA, et al. The economic burden of vision loss and blindness in the United States. Ophthalmology. 2022;129:369–78. 34560128 10.1016/j.ophtha.2021.09.010

[10] Yang S, Zhao J, Sun X. Resistance to anti-VEGF therapy in neovascular age-related macular degeneration: a comprehensive review. Drug Des Devel Ther. 2016;10:1857–67. 27330279 10.2147/DDDT.S97653 PMC4898027

[11] Cui QN, Gray IN, Yu Y, VanderBeek BL. Repeated intravitreal injections of antivascular endothelial growth factors and risk of intraocular pressure medication use. Graefes Arch Clin Exp Ophthalmol. 2019;257:1931–9. 31152311 10.1007/s00417-019-04362-7 PMC6698200

[12] Raguram A, Banskota S, Liu DR. Therapeutic in vivo delivery of gene editing agents. Cell. 2022;185:2806–27. 35798006 10.1016/j.cell.2022.03.045 PMC9454337

[13] Horvath P, Barrangou R. CRISPR/Cas, the immune system of bacteria and archaea. Science. 2010;327:167–70. 20056882 10.1126/science.1179555

[14] Wiedenheft B, Sternberg SH, Doudna JA. RNA-guided genetic silencing systems in bacteria and archaea. Nature. 2012;482:331–8. 22337052 10.1038/nature10886

[15] Gaudelli NM, Komor AC, Rees HA, Packer MS, Badran AH, Bryson DI, et al. Programmable base editing of A•T to G•C in genomic DNA without DNA cleavage. Nature. 2017;551:464–71. 29160308 10.1038/nature24644 PMC5726555

[16] Komor AC, Kim YB, Packer MS, Zuris JA, Liu DR. Programmable editing of a target base in genomic DNA without double-stranded DNA cleavage. Nature. 2016;533:420–4. 27096365 10.1038/nature17946 PMC4873371

[17] Anzalone AV, Randolph PB, Davis JR, Sousa AA, Koblan LW, Levy JM, et al. Search-and-replace genome editing without double-strand breaks or donor DNA. Nature. 2019;576:149–57. 31634902 10.1038/s41586-019-1711-4 PMC6907074

[18] Wang D, Tai PWL, Gao G. Adeno-associated virus vector as a platform for gene therapy delivery. Nat Rev Drug Discov. 2019;18:358–78. 30710128 10.1038/s41573-019-0012-9 PMC6927556

[19] Pierce EA, Bennett J. The status of RPE65 gene therapy trials: safety and efficacy. Cold Spring Harb Perspect Med. 2015;5:a017285. 25635059 10.1101/cshperspect.a017285 PMC4561397

[20] Testa F, Maguire AM, Rossi S, et al. Three-year follow-up after unilateral subretinal delivery of adeno-associated virus in patients with Leber congenital amaurosis type 2. Ophthalmology. 2013;120:1283–91. 23474247 10.1016/j.ophtha.2012.11.048 PMC3674112

[21] Suh S, Choi EH, Raguram A, Liu DR, Palczewski K. Precision genome editing in the eye. Proc Natl Acad Sci U S A. 2022;119:e2210104119. 36122230 10.1073/pnas.2210104119 PMC9522375

[22] Biswal MR, Prentice HM, Smith GW, Zhu P, Tong Y, Dorey CK, et al. Cell-specific gene therapy driven by an optimized hypoxia-regulated vector reduces choroidal neovascularization. J Mol Med (Berl). 2018;96:1107–18. 30105447 10.1007/s00109-018-1683-0

[23] Koo T, Park SW, Jo DH, Kim D, Kim JH, Cho HY, et al. CRISPR-Lbcpf1 prevents choroidal neovascularization in a mouse model of age-related macular degeneration. Nat Commun. 2018;9:1855. 29748595 10.1038/s41467-018-04175-y PMC5945874

[24] Wang JH, Roberts GE, Liu GS. Updates on gene therapy for diabetic retinopathy. Curr Diab Rep. 2020;20:22. 32415508 10.1007/s11892-020-01308-w PMC7228867

[25] Bucher K, Rodríguez-Bocanegra E, Dauletbekov D, Fischer MD. Immune responses to retinal gene therapy using adeno-associated viral vectors - implications for treatment success and safety. Prog Retin Eye Res. 2021;83:100915. 33069860 10.1016/j.preteyeres.2020.100915

[26] Wei T, Cheng Q, Min YL, Olson EN, Siegwart DJ. Systemic nanoparticle delivery of CRISPR-Cas9 ribonucleoproteins for effective tissue specific genome editing. Nat Commun. 2020;11:3232. 32591530 10.1038/s41467-020-17029-3 PMC7320157

[27] Loughrey D, Dahlman JE. Non-liver mRNA delivery. Acc Chem Res. 2022;55:13–23. 34859663 10.1021/acs.accounts.1c00601

[28] Paunovska K, Loughrey D, Dahlman JE. Drug delivery systems for RNA therapeutics. Nat Rev Genet. 2022;23:265–80. 34983972 10.1038/s41576-021-00439-4 PMC8724758

[29] Kent SJ, Li S, Amarasena TH, et al. Blood distribution of SARS-CoV-2 lipid nanoparticle mRNA vaccine in humans. ACS Nano. 2024;18:27077–89. 39298422 10.1021/acsnano.4c11652 PMC11447892

[30] Li W, Vanluchene H, Raes L, et al. Efficacy versus immunogenicity of LNP-mediated delivery of mRNA and self-amplifying RNA upon intravitreal injection in the mouse eye. J Control Release. 2025;385:114027. 40659060 10.1016/j.jconrel.2025.114027

[31] Lyu P, Javidi-Parsijani P, Atala A, Lu B. Delivering Cas9/sgRNA ribonucleoprotein (RNP) by lentiviral capsid-based bionanoparticles for efficient “hit-and-run” genome editing. Nucleic Acids Res. 2019;47:e99. 31299082 10.1093/nar/gkz605 PMC6753487

[32] Lyu P, Lu Z, Cho SI, Yadav M, Yoo KW, Atala A, et al. Adenine base editor ribonucleoproteins delivered by lentivirus-like particles show high on-target base editing and undetectable RNA off-target activities. CRISPR J. 2021;4:69–81. 33616436 10.1089/crispr.2020.0095

[33] Nooraei S, Bahrulolum H, Hoseini ZS, Katalani C, Hajizade A, Easton AJ, et al. Virus-like particles: preparation, immunogenicity and their roles as nanovaccines and drug nanocarriers. J Nanobiotechnology. 2021;19:59. 33632278 10.1186/s12951-021-00806-7 PMC7905985

[34] Campbell LA, Coke LM, Richie CT, Fortuno LV, Park AY, Harvey BK. Gesicle-mediated delivery of CRISPR/Cas9 ribonucleoprotein complex for inactivating the HIV provirus. Mol Ther. 2019;27:151–63. 30389355 10.1016/j.ymthe.2018.10.002 PMC6318701

[35] Choi JG, Dang Y, Abraham S, Ma H, Zhang J, Guo H, et al. Lentivirus pre-packed with Cas9 protein for safer gene editing. Gene Ther. 2016;23:627–33. 27052803 10.1038/gt.2016.27

[36] Hamilton JR, Tsuchida CA, Nguyen DN, Shy BR, McGarrigle ER, Sandoval Espinoza CR, et al. Targeted delivery of CRISPR-Cas9 and transgenes enables complex immune cell engineering. Cell Rep. 2021;35:109207. 34077734 10.1016/j.celrep.2021.109207 PMC8236216

[37] Banskota S, Raguram A, Suh S, et al. Engineered virus-like particles for efficient in vivo delivery of therapeutic proteins. Cell. 2022;185:250-265.e216. 35021064 10.1016/j.cell.2021.12.021 PMC8809250

[38] Lambert V, Lecomte J, Hansen S, et al. Laser-induced choroidal neovascularization model to study age-related macular degeneration in mice. Nat Protoc. 2013;8:2197–211. 24136346 10.1038/nprot.2013.135

[39] Salas A, Badia A, Fontrodona L, Zapata M, García-Arumí J, Duarri A. Neovascular progression and retinal dysfunction in the laser-induced choroidal neovascularization mouse model. Biomedicines. 2023. 10.3390/biomedicines11092445. 37760886 10.3390/biomedicines11092445 PMC10525599

[40] Kim K, Park SW, Kim JH, Lee SH, Kim D, Koo T, et al. Genome surgery using Cas9 ribonucleoproteins for the treatment of age-related macular degeneration. Genome Res. 2017;27:419–26. 28209587 10.1101/gr.219089.116 PMC5340969

[41] Stemmer M, Thumberger T, Del Sol Keyer M, Wittbrodt J, Mateo JL. Cctop: an intuitive, flexible and reliable CRISPR/Cas9 target prediction tool. PLoS ONE. 2015;10:e0124633. 25909470 10.1371/journal.pone.0124633 PMC4409221

[42] Pan D, Gunther R, Duan W, Wendell S, Kaemmerer W, Kafri T, et al. Biodistribution and toxicity studies of VSVG-pseudotyped lentiviral vector after intravenous administration in mice with the observation of in vivo transduction of bone marrow. Mol Ther. 2002;6:19–29. 12095299 10.1006/mthe.2002.0630

[43] Chen ST, Iida A, Guo L, Friedmann T, Yee JK. Generation of packaging cell lines for pseudotyped retroviral vectors of the G protein of vesicular stomatitis virus by using a modified tetracycline inducible system. Proc Natl Acad Sci U S A. 1996;93:10057–62. 8816750 10.1073/pnas.93.19.10057 PMC38335

[44] Kim E, Koo T, Park SW, et al. In vivo genome editing with a small Cas9 orthologue derived from *Campylobacter jejuni*. Nat Commun. 2017;8:14500. 28220790 10.1038/ncomms14500 PMC5473640

[45] Haldrup J, Andersen S, Labial ARL, et al. Engineered lentivirus-derived nanoparticles (LVNPs) for delivery of CRISPR/Cas ribonucleoprotein complexes supporting base editing, prime editing and in vivo gene modification. Nucleic Acids Res. 2023;51:10059–74. 37678882 10.1093/nar/gkad676 PMC10570023

[46] Collins FS, Varmus H. A new initiative on precision medicine. N Engl J Med. 2015;372:793–5. 25635347 10.1056/NEJMp1500523 PMC5101938

[47] Feero WG, Wicklund CA, Veenstra D. Precision medicine, genome sequencing, and improved population health. JAMA. 2018;319:1979–80. 29547675 10.1001/jama.2018.2925

[48] Ikwuagwu B, Tullman-Ercek D. Virus-like particles for drug delivery: a review of methods and applications. Curr Opin Biotechnol. 2022;78:102785. 36099859 10.1016/j.copbio.2022.102785

[49] Mangeot PE, Risson V, Fusil F, et al. Genome editing in primary cells and in vivo using viral-derived nanoblades loaded with Cas9-sgRNA ribonucleoproteins. Nat Commun. 2019;10:45. 30604748 10.1038/s41467-018-07845-z PMC6318322

[50] Lyu P, Wang L, Lu B. Virus-like particle mediated CRISPR/Cas9 delivery for efficient and safe genome editing. Life. 2020. 10.3390/life10120366. 33371215 10.3390/life10120366 PMC7766694

[51] An M, Raguram A, Du SW, Banskota S, Davis JR, Newby GA, et al. Engineered virus-like particles for transient delivery of prime editor ribonucleoprotein complexes in vivo. Nat Biotechnol. 2024;42:1526–37. 38191664 10.1038/s41587-023-02078-y PMC11228131

[52] Ling S, Zhang X, Dai Y, et al. Customizable virus-like particles deliver CRISPR-Cas9 ribonucleoprotein for effective ocular neovascular and Huntington’s disease gene therapy. Nat Nanotechnol. 2025;20:543–53. 39930103 10.1038/s41565-024-01851-7 PMC12015117

[53] Jang H, Jo DH, Cho CS, Shin JH, Seo JH, Yu G, et al. Application of prime editing to the correction of mutations and phenotypes in adult mice with liver and eye diseases. Nat Biomed Eng. 2022;6:181–94. 34446856 10.1038/s41551-021-00788-9

[54] Alsing S, Lindholm AB, Haldrup J, Jensen EG, Mikkelsen JG, Aagaard L, Askou AL, Corydon T. Simple autofluorescence-restrictive sorting of eGFP+ RPE cells allows reliable assessment of targeted retinal gene therapy. Front Drug Delivery. 2022;2.

[55] L’Abbate D, Prescott K, Geraghty B, Kearns VR, Steel DHW. Biomechanical considerations for optimising subretinal injections. Surv Ophthalmol. 2024;69:722–32. 38797394 10.1016/j.survophthal.2024.05.004

[56] Qi Y, Dai X, Zhang H, et al. Trans-corneal subretinal injection in mice and its effect on the function and morphology of the retina. PLoS ONE. 2015;10:e0136523. 26317758 10.1371/journal.pone.0136523 PMC4552822

[57] Pulman J, Botto C, Malki H, et al. Direct delivery of Cas9 or base editor protein and guide RNA complex enables genome editing in the retina. Mol Ther Nucleic Acids. 2024;35:102349. 39494148 10.1016/j.omtn.2024.102349 PMC11531619

[58] Kurihara T, Westenskow PD, Bravo S, Aguilar E, Friedlander M. Targeted deletion of *Vegfa* in adult mice induces vision loss. J Clin Invest. 2012;122:4213–7. 23093773 10.1172/JCI65157 PMC3484459

[59] Wang D, Zhang F, Gao G. CRISPR-based therapeutic genome editing: strategies and in vivo delivery by AAV vectors. Cell. 2020;181:136–50. 32243786 10.1016/j.cell.2020.03.023 PMC7236621

[60] Pupo A, Fernández A, Low SH, François A, Suárez-Amarán L, Samulski RJ. AAV vectors: the Rubik’s cube of human gene therapy. Mol Ther. 2022;30:3515–41. 36203359 10.1016/j.ymthe.2022.09.015 PMC9734031

[61] Wills CA, Drago D, Pietrusko RG. Clinical holds for cell and gene therapy trials: risks, impact, and lessons learned. Mol Ther Methods Clin Dev. 2023;31:101125. 37886603 10.1016/j.omtm.2023.101125 PMC10597781

[62] Li W, Asokan A, Wu Z, et al. Engineering and selection of shuffled AAV genomes: a new strategy for producing targeted biological nanoparticles. Mol Ther. 2008;16:1252–60. 18500254 10.1038/mt.2008.100 PMC2632803

[63] Bainbridge JW, Mehat MS, Sundaram V, et al. Long-term effect of gene therapy on Leber’s congenital amaurosis. N Engl J Med. 2015;372:1887–97. 25938638 10.1056/NEJMoa1414221 PMC4497809

[64] Jacobson SG, Cideciyan AV, Roman AJ, Sumaroka A, Schwartz SB, Heon E, et al. Improvement and decline in vision with gene therapy in childhood blindness. N Engl J Med. 2015;372:1920–6. 25936984 10.1056/NEJMoa1412965 PMC4450362

[65] Renner TM, Tang VA, Burger D, Langlois MA. Intact viral particle counts measured by flow virometry provide insight into the infectivity and genome packaging efficiency of moloney murine leukemia virus. J Virol. 2020. 10.1128/JVI.01600-19. 31694951 10.1128/JVI.01600-19 PMC6955258

[66] Park J, Lim K, Kim JS, Bae S. Cas-analyzer: an online tool for assessing genome editing results using NGS data. Bioinformatics. 2017;33:286–8. 27559154 10.1093/bioinformatics/btw561 PMC5254075

[67] Kim D, Bae S, Park J, Kim E, Kim S, Yu HR, Hwang J, Kim JI, Kim JS. Digenome-seq: genome-wide profiling of CRISPR-Cas9 off-target effects in human cells. Nat Methods. 2015;12**:**237–243, 231 p following 243. 10.1038/nmeth.3284 25664545

[68] Kim D, Kim J, Hur JK, Been KW, Yoon SH, Kim JS. Genome-wide analysis reveals specificities of Cpf1 endonucleases in human cells. Nat Biotechnol. 2016;34:863–8. 27272384 10.1038/nbt.3609

[69] Wu J, Jo DH, Fruttiger M, Kim JH. Cone cell dysfunction attenuates retinal neovascularization in oxygen-induced retinopathy mouse model. J Neurosci Res. 2024;102:e25316. 38415926 10.1002/jnr.25316

[70] Wu J, Jang H, Kwak H, Son M, Jiang W, Hwang HY, Jo DH, Kim D, Kim HH, Kim JH. VLP-assembled Vegfa-targeting Cas9 ribonucleoprotein treatment alleviates neovascularization in wAMD mice. Bioproject accession: PRJNA1117205. 2024. https://www.ncbi.nlm.nih.gov/bioproject/PRJNA1117205. 10.1186/s13059-025-03774-5 PMC12509411 41068986

[71] Wu J, Jang H, Kwak H, Son M, Jiang W, Hwang HY, et al. Engineered virus-like particle-assembled Vegfa-targeting Cas9 ribonucleoprotein treatment alleviates neovascularization in wet age-related macular degeneration. 2025. Zenodo. 10.5281/zenodo.17019773.2025. 10.1186/s13059-025-03774-5 PMC12509411 41068986

