# Supplementary material for: Engineered virus-like particle-assembled Vegfa-targeting Cas9 ribonucleoprotein treatment alleviates neovascularization in wet age-related macular degeneration
Source: Genome Biol. 2025 Oct 9;26:346. doi: 10.1186/s13059-025-03774-5 (PMC12509411; doi:10.1186/s13059-025-03774-5)
Supplement: Supplementary file 1 — Additional file 1: Fig. S1. The full gels of Western blotting data in supplementary information. Fig. S2. Cas9-eVLPs delivered subretinally are localized to the injection site. Fig. S3. Indel frequencies from whole RPE tissue. Fig. S4. Indel frequencies from mouse retina. Fig. S5. Excessive delivery of Cas9-eVLPs induces retinal degeneration in mice. Fig. S6. Recovery process of artificially induced retinal detachment after subretinal injection. Fig. S7. Evaluate the toxicity of Cas9-eVLPs by comparing the retinal anatomical structures. Fig. S8. Detection of retinal apoptotic cells by TUNEL assay. Fig. S9. Detection of activation of immune cells in retina. [file 13059_2025_3774_MOESM1_ESM.pdf]

Additional file 1

Article title: Engineered virus-like particle-assembled *Vegfa*-targeting Cas9 ribonucleoprotein treatment alleviates neovascularization in wet age-related macular degeneration

Author name: Jun Wu, Hyewon Jang, Hyunjong Kwak, Minchae Son, Weiyan Jiang, Hye-Yeon Hwang, Dong Hyun Jo, Daesik Kim, Hyongbum Henry Kim, and Jeong Hun Kim

Correspondence author:
Hyongbum Henry Kim, MD, PhD
Department of Pharmacology, Yonsei University College of Medicine, Seoul 03722, Republic of Korea.
E-mail address: hkim1@yuhs.ac, aquamd@gmail.com

Jeong Hun Kim, MD, PhD
Department of Biomedical Sciences & Ophthalmology, Seoul National University College of Medicine, Seoul 03080, Republic of Korea.
E-mail address: steph25@snu.ac.kr

Table of contents

Fig. S1 The full gels of Western blot data in supplementary information.
Fig. S2 Cas9-eVLPs delivered subretinally are localized to the injection site.
Fig. S3 Indel frequencies from whole RPE tissue.
Fig. S4 Indel frequencies from mouse retina.
Fig. S5 Excessive delivery of Cas9-eVLPs induces retinal degeneration in mice.
Fig. S6 Recovery process of artificially induced retinal detachment after subretinal injection.
Fig. S7 Evaluate the toxicity of Cas9-eVLPs by comparing the retinal anatomical structures.
Fig. S8 Detection of retinal apoptotic cells by TUNEL assay.
Fig. S9 Detection of activation of immune cells in retina.

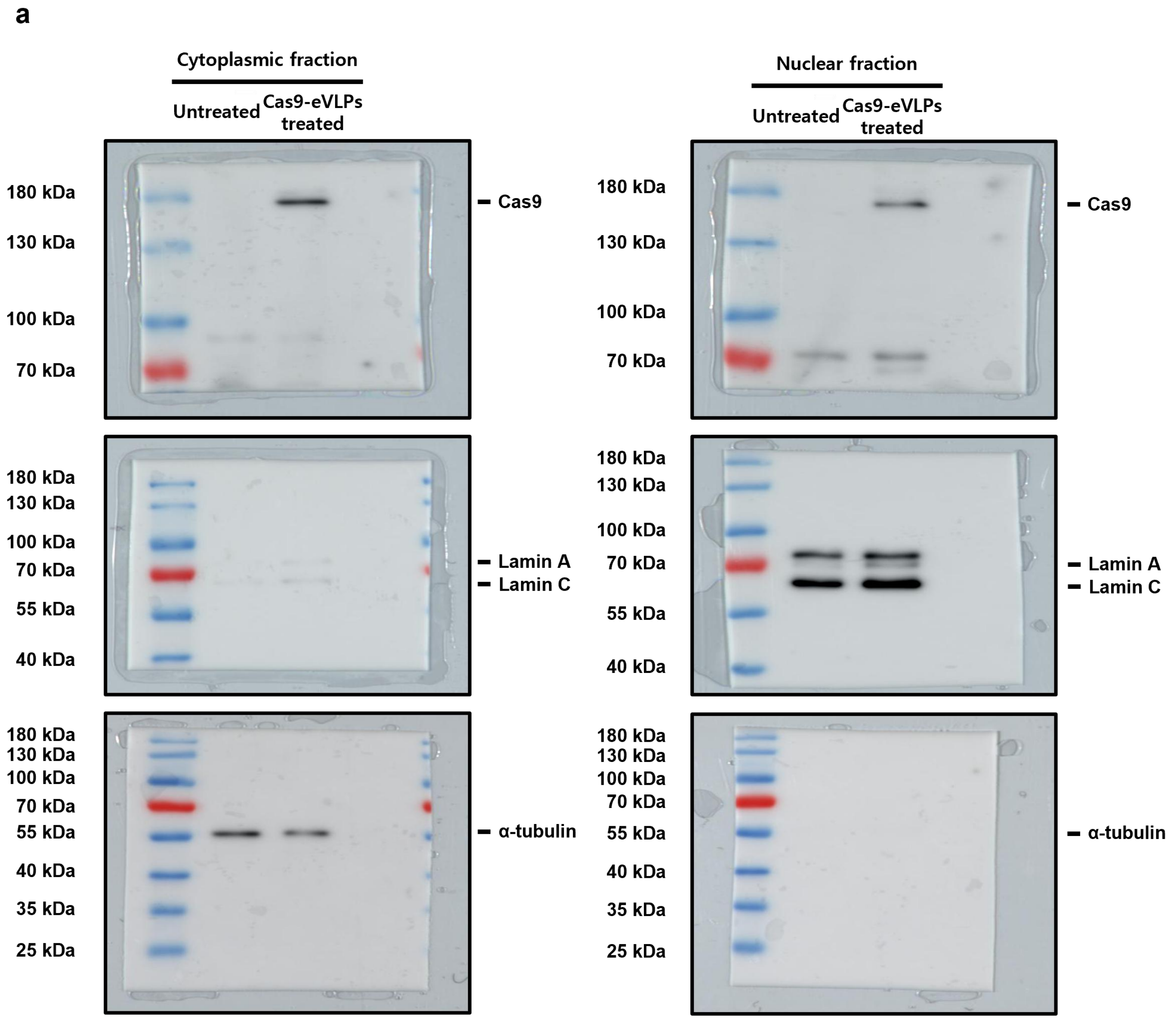

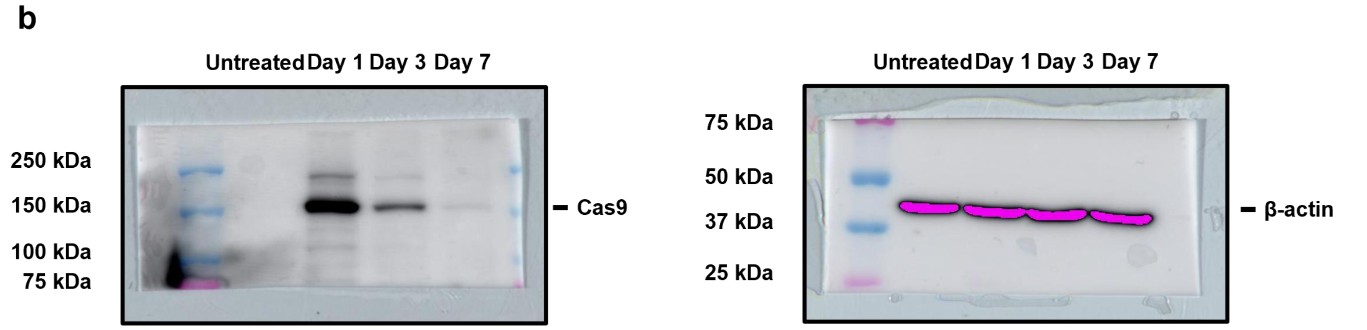

Fig. S1 The full gels of Western blot data in supplementary information.

a The full Western blotting images for Fig. 1d. **b** The full Western blotting images for Fig. 2c.

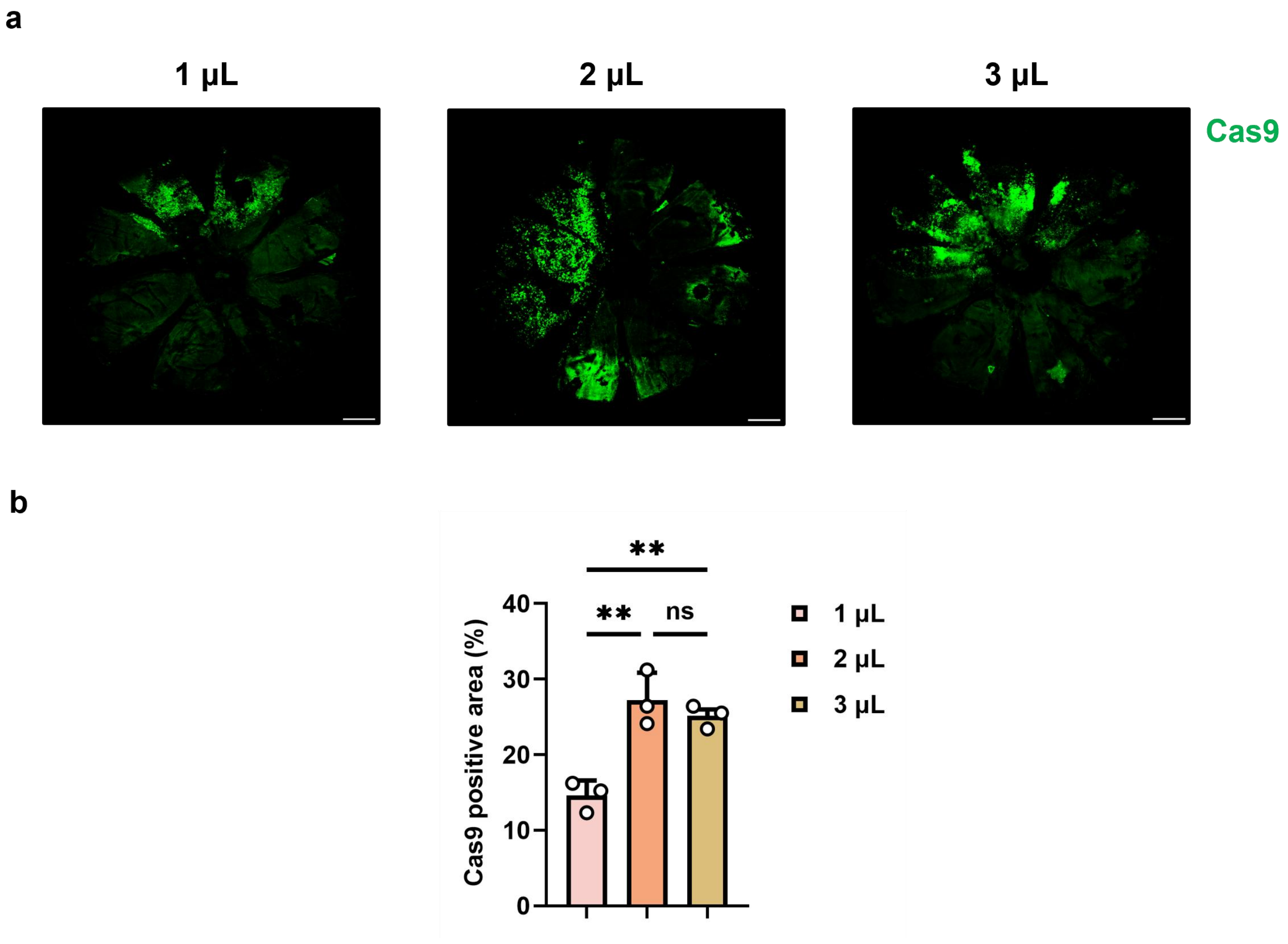

Fig. S2 Cas9-eVLPs delivered subretinally are localized to the injection site.

a Representative immunofluorescence images of RCS complexes after 1 day subretinal injection of Cas9-eVLPs using different injection volumes. Cas9 (Green). Scale bar: 500 μm. **b** Quantification of Cas9-positive area of the whole RCS tissue (n = 3). Data are presented as mean ± SD. Statistical analyses were done using one-way ANOVA followed by Tukey's *post hoc* multiple comparison tests. ns, not significant. $^{**}p < 0.01$.

a

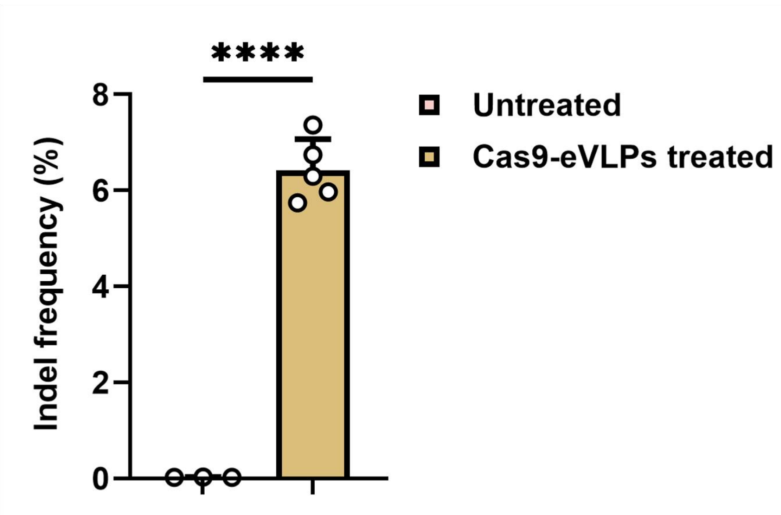

Fig. S3. Indel frequencies from whole RPE tissue.

a Indel frequencies induced *in vivo* determined using genomic DNA isolated from the entire RPE tissue after subretinal injection of 4.3×10^{10} Cas9-eVLPs (n = 3 for untreated group, n = 5 for Cas9-eVLPs treated group). Data are presented as mean ± SD. Statistical analyses were done using Student's t test. ****$p < 0.0001$.

a

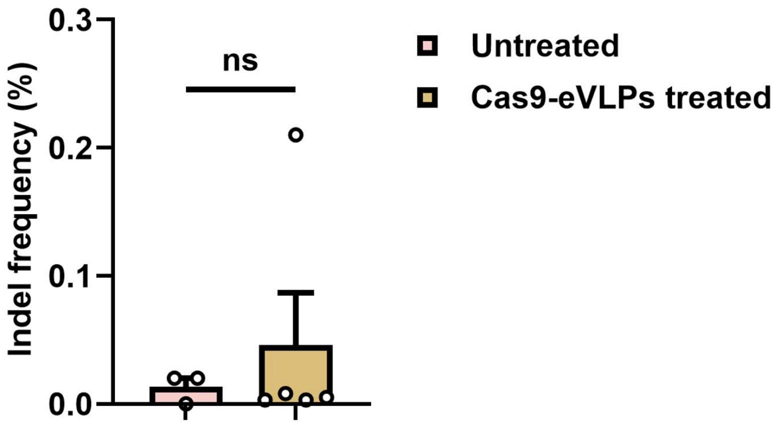

Fig. S4 Indel frequencies from mouse retina.

a Indel frequencies induced *in vivo* determined using genomic DNA isolated from the mouse retina after subretinal injection of 4.3 × 10^{10} Cas9-eVLPs (n = 3 for untreated group, n = 5 for Cas9-eVLPs treated group). Data are presented as mean ± SD. Statistical analyses were done using Student's t test. ns, not significant.

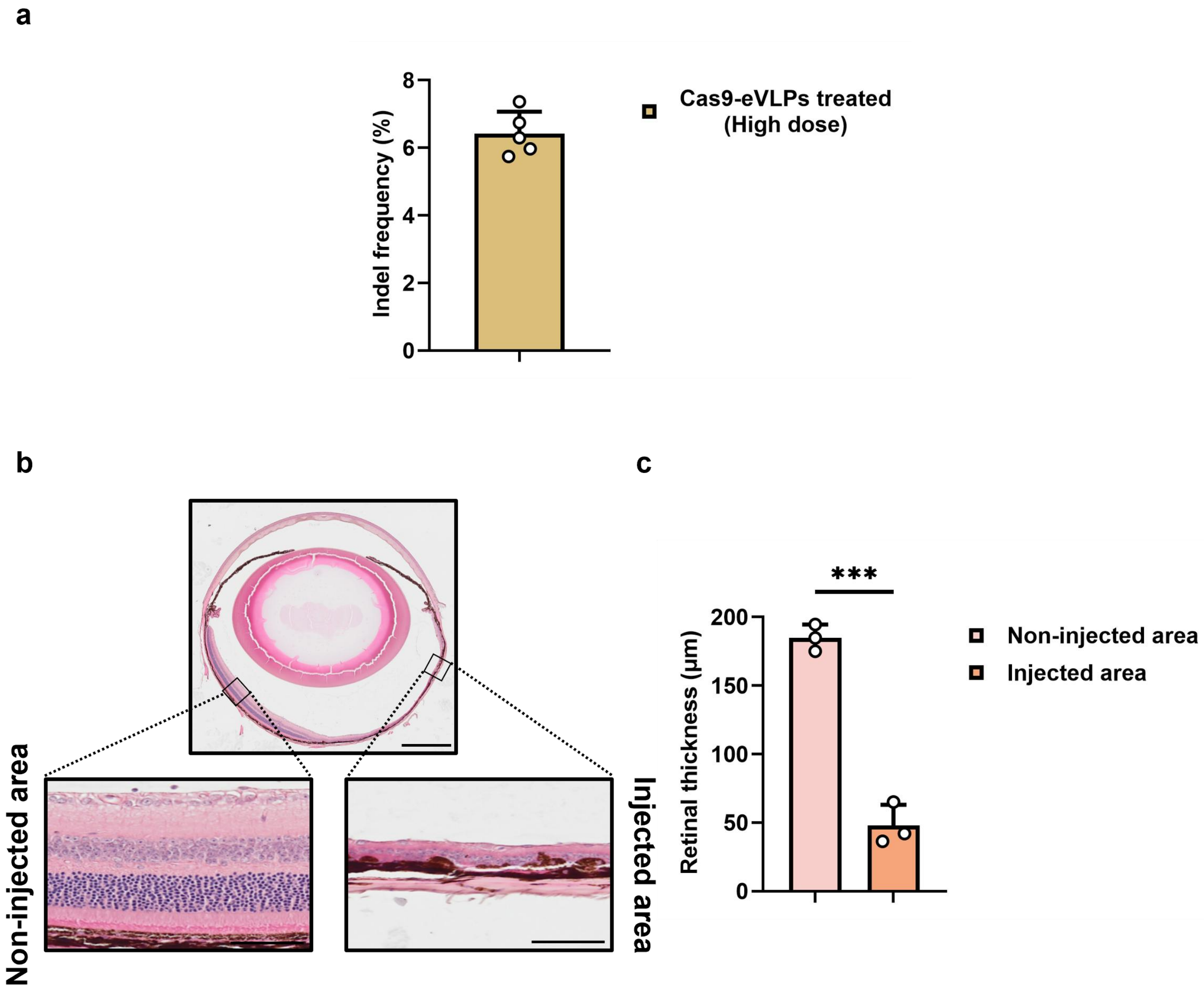

Fig. S5. Excessive delivery of Cas9-eVLPs induces retinal degeneration in mice.

a Indel frequencies in the injected area of RPE tissue 7 days after subretinal injection of 2.15×10^{11} Cas9-eVLPs (n = 5). **b** H&E staining images of high dose Cas9-eVLPs treatment after 4 weeks of injection. Scale bar: 500 μm (top), 50 μm (bottom). **c** Quantification of retinal thickness in proximity to the subretinal injected site and non-injected site (n = 3). Data are presented as mean ± SD. Statistical analyses were done using Student's t test. ***$p < 0.001$.

a

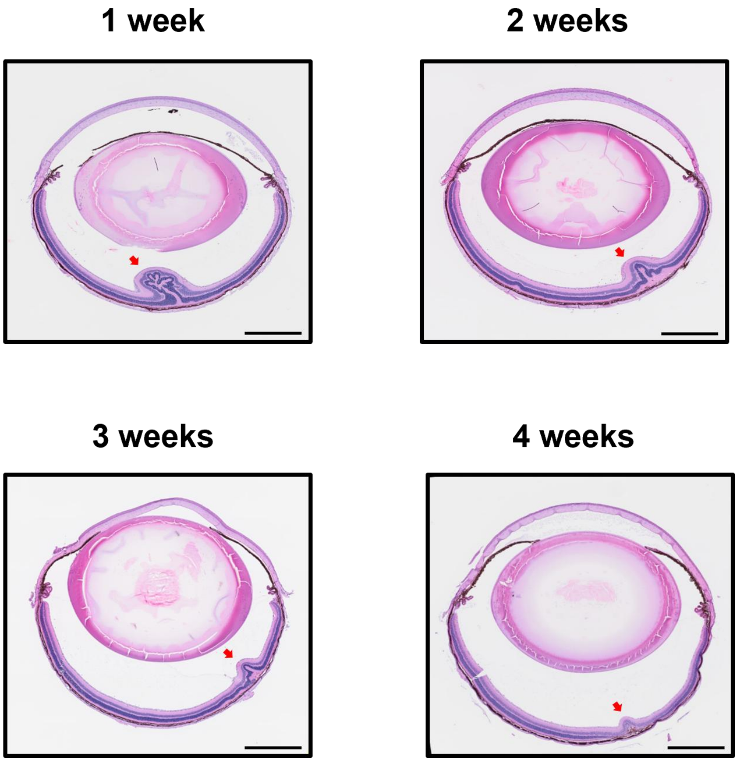

Fig. S6 Recovery process of artificially induced retinal detachment after subretinal injection.

a After 2 µL of Cas9-eVLPs was injected into the subretinal space, the retinal detachment gradually recovered over time. Four weeks after the injection, the detached retina was almost flat. Scale bar: 500 µm.

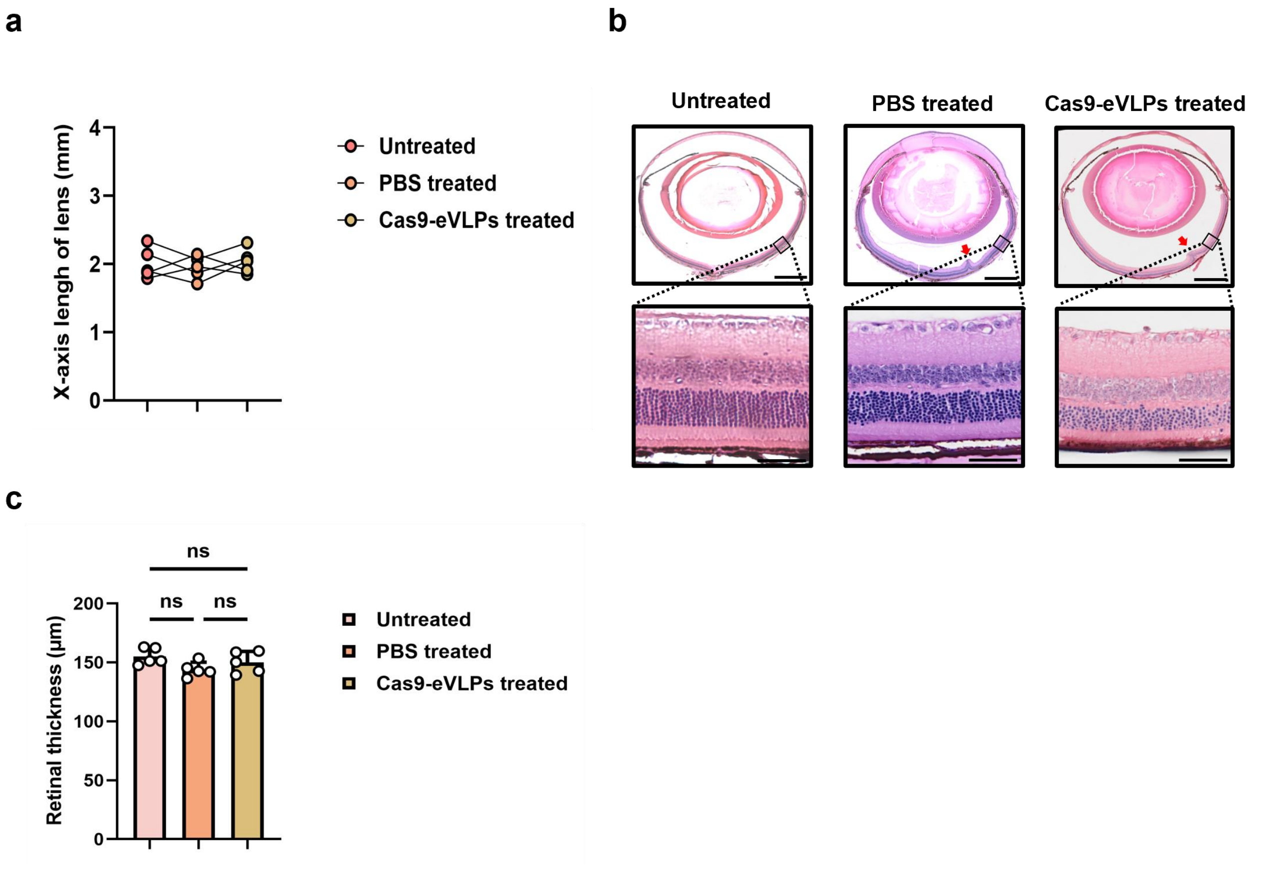

Fig. S7 Evaluate the toxicity of Cas9-eVLPs by comparing the retinal anatomical structures.

a Determine the cross-section position of the eyeball by comparing the X-axis length of lens. **b** Representative H&E staining images after 4 weeks of injection. Red arrows indicate subretinal injection site. The region marked by the black box was enlarged and displayed below. Scale bar: 500 μm (top), 50 μm (bottom). **c** Quantification of retinal thickness of untreated, PBS treated and Cas9-eVLPs treated mice (n = 5). Data are presented as mean ± SD. Statistical analyses were done using one-way ANOVA followed by Tukey's *post hoc* multiple comparison tests. ns, not significant.

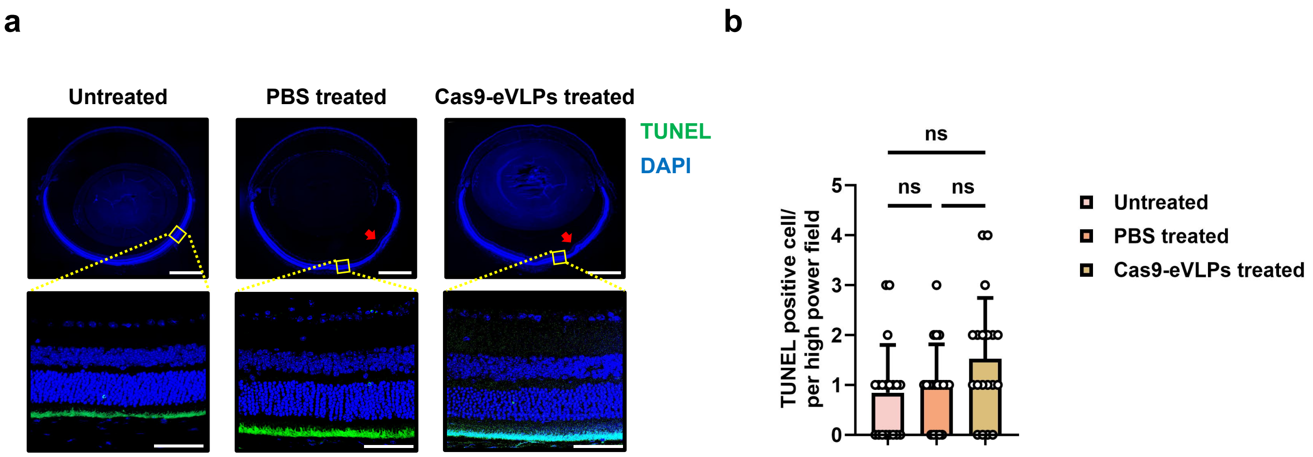

Fig. S8. Detection of retinal apoptotic cells by TUNEL assay.

a Representative micrographs of retinal sections evaluated for apoptosis using the TUNEL assay. Red arrows indicate subretinal injection site. The region marked by the yellow box was enlarged and displayed below. Scale bar: 500 µm (top), 50 µm (bottom). **b** Quantification of TUNEL-positive cells in each group (n = 20). Data are presented as mean ± SD. Statistical analyses were done using One-way ANOVA and Tukey's *post hoc* multiple comparison tests.

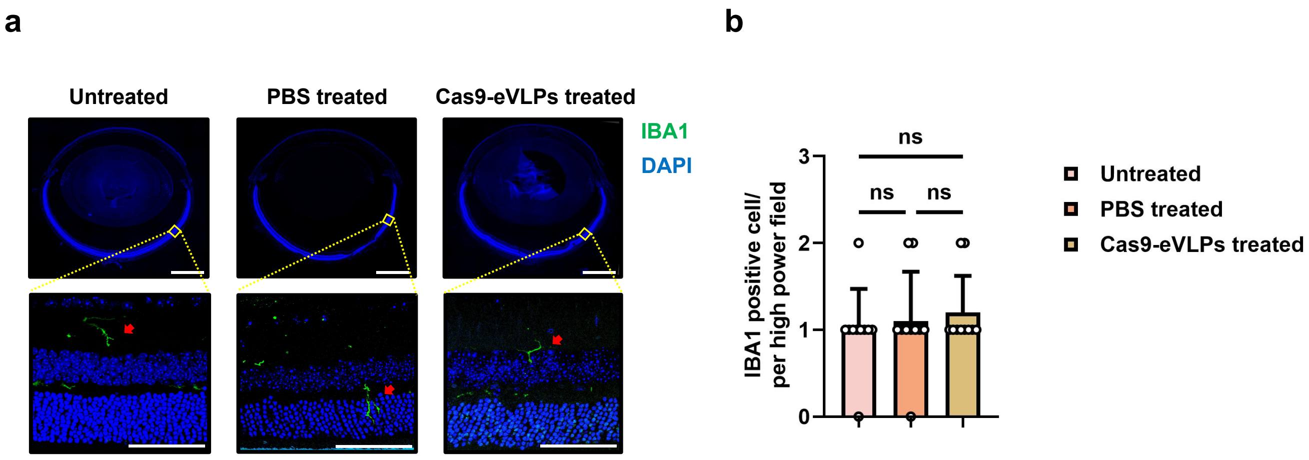

Fig. S9. Detection of activation of immune cells in retina.

a Representative retinal cross sections of all groups stained with DAPI (blue) and microglia marker-IBA1 (green). The region marked by the yellow box was enlarged and displayed below. Red arrows indicate ramified microglia. Scale bar: 500 µm (top), 50 µm (bottom). **b** Quantification data showing the number of cells per section post intervention for IBA1-positive cells (n = 10). Data are presented as mean ± SD. Statistical analyses were done using One-way ANOVA and Tukey's *post hoc* multiple comparison tests.
